# Impact of Sarcoplasmic Reticulum Calcium Release on Calcium Dynamics and Action Potential Morphology in Human Atrial Myocytes: A Computational Study

**DOI:** 10.1371/journal.pcbi.1001067

**Published:** 2011-01-27

**Authors:** Jussi T. Koivumäki, Topi Korhonen, Pasi Tavi

**Affiliations:** Department of Biotechnology and Molecular Medicine, A.I. Virtanen Institute for Molecular Sciences, University of Eastern Finland, Kuopio, Finland; University of California San Diego, United States of America

## Abstract

Electrophysiological studies of the human heart face the fundamental challenge that experimental data can be acquired only from patients with underlying heart disease. Regarding human atria, there exist sizable gaps in the understanding of the functional role of cellular Ca^2+^ dynamics, which differ crucially from that of ventricular cells, in the modulation of excitation-contraction coupling. Accordingly, the objective of this study was to develop a mathematical model of the human atrial myocyte that, in addition to the sarcolemmal (SL) ion currents, accounts for the heterogeneity of intracellular Ca^2+^ dynamics emerging from a structurally detailed sarcoplasmic reticulum (SR). Based on the simulation results, our model convincingly reproduces the principal characteristics of Ca^2+^ dynamics: 1) the biphasic increment during the upstroke of the Ca^2+^ transient resulting from the delay between the peripheral and central SR Ca^2+^ release, and 2) the relative contribution of SL Ca^2+^ current and SR Ca^2+^ release to the Ca^2+^ transient. In line with experimental findings, the model also replicates the strong impact of intracellular Ca^2+^ dynamics on the shape of the action potential. The simulation results suggest that the peripheral SR Ca^2+^ release sites define the interface between Ca^2+^ and AP, whereas the central release sites are important for the fire-diffuse-fire propagation of Ca^2+^ diffusion. Furthermore, our analysis predicts that the modulation of the action potential duration due to increasing heart rate is largely mediated by changes in the intracellular Na^+^ concentration. Finally, the results indicate that the SR Ca^2+^ release is a strong modulator of AP duration and, consequently, myocyte refractoriness/excitability. We conclude that the developed model is robust and reproduces many fundamental aspects of the tight coupling between SL ion currents and intracellular Ca^2+^ signaling. Thus, the model provides a useful framework for future studies of excitation-contraction coupling in human atrial myocytes.

## Author Summary

In the human heart, the contraction of atrial and ventricular muscle cells is based largely on common mechanisms. There is, however, a fundamental difference in the cellular calcium dynamics that underlie the contractile function. Here, we have developed a computational model of the human atrial cell that convincingly reproduces the experimentally observed characteristics of the electrical activity and the cyclic fluctuations of the intracellular calcium concentration. With the model, we evaluate the relative roles of the most important cellular calcium transport mechanisms and their impact on the electrical behavior of the cell. Our simulations predict that the amount of calcium released from the cellular stores during each electrical cycle crucially regulates the excitability of the human atrial cell. Furthermore, the results indicate that the cellular sodium accumulation related to faster heart rates is one of the main mechanisms driving the adaptation of cardiac electrical activity. Finally, we conclude that the presented model also provides a useful framework for future studies of human atrial cells.

## Introduction

In cardiac myocytes, the process triggered by the action potential (AP) and resulting in the contraction of the myocyte is commonly referred to as excitation-contraction coupling (ECC) [1]. The transient elevation of intracellular Ca^2+^ concentration ([Ca^2+^]_i_) that underlies the contraction is initiated by the Ca^2+^ influx from the extracellular space through the L-type calcium channels (LTCCs), which causes the release of more Ca^2+^ from the sarcoplasmic reticulum (SR) via the SR calcium release channels (ryanodine receptors; RyRs), This mechanism is known as Ca^2+^ induced Ca^2+^ release (CICR) [2]. During one contraction cycle, the Ca^2+^ influx has to be balanced with an efflux to the same compartments, in order that Ca^2+^does not start to accumulate and impede contraction. The majority of Ca^2+^ is re-circulated back to the SR by the SR Ca^2+^-ATPase (SERCA), leaving a smaller fraction of Ca^2+^ to be extruded from the cell by the Na^+^/Ca^2+^ exchanger (NCX) and plasmalemmal Ca^2+^-ATPase (PMCA).

Whilst the same CICR mechanism initiates the transient elevation of [Ca^2+^]_i_ in both ventricular and atrial myocytes, there are substantial spatiotemporal differences in the properties of the atrial and ventricular Ca^2+^ transients [3], [4] due to the divergent intracellular ultrastructures. Mammalian atrial myocytes lack a prominent transverse tubular system [5], which in ventricular myocytes establishes the tight coupling of the SR to sarcolemma, enabling a Ca^2+^ release that is virtually uniform throughout the cell [6]. In atrial myocytes, however, the Ca^2+^ wave arises in the periphery (junctional-SR) and then propagates to the center of the cell, activating secondary release from the corbular (non-junctional) SR compartments [3], [7], [8].

Facing the complexity of a highly integrated and interdependent system, mathematical modeling has become an established complement to the experimental approach in elucidation of the mechanisms that underlie cardiac electrophysiology [9]. In human studies, the role of mathematical modeling is perhaps even more important because there are substantial limitations in the quantity and quality of the human cardiac tissue that is available for *in vitro* experiments. In this study, we present a model of the adult human atrial myocyte that has a spatially detailed and physiologically based formulation of the Ca^2+^ release from and uptake to the SR. Based on a realistic description of the interrelations between [Ca^2+^]_i_, sarcolemmal (SL) ion currents, and the SR Ca^2+^ release, the aim of this study was to elucidate to what extent intracellular Ca^2+^ regulates the AP waveform, cellular excitability, and rate-dependent electrophysiological mechanisms. The results indicate that the peripheral (junctional) SR Ca^2+^ release sites define the interface between Ca^2+^ and AP, whereas the central (non-junctional) release sites are important for the fire-diffuse-fire propagation of Ca^2+^ diffusion. Moreover, our analysis suggests that the rising intracellular Na^+^ concentration is an important modulator of the action potential duration at increasing heart rates. Finally, the results predict that SR Ca^2+^ release is a strong modulator of AP duration and thus affects the human atrial myocyte refractoriness.

## Methods

The presented mathematical model is a set of ordinary differential equations that were implemented to the Matlab (The MathWorks) environment of technical programming. A brief description of the novel and modified model components are given below. The complete mathematical formulation of the model and parameters are shown in Text S1, and initial values of the differential variables in Table S4 in the Supporting Information.

Simulation results were obtained by numerically integrating the model equations with a stiff ordinary differential equation solver method (ode15s). Despite the detailed description of intracellular Ca^2+^ dynamics, the computational load of the model is rather low: a 10-second simulation at 1 Hz pacing takes about 10 seconds to run on a normal desktop PC (Intel Core Duo CPU, 2.8 GHz, 2 GB of RAM).

### Cellular structure of the model

**Figure 1:**
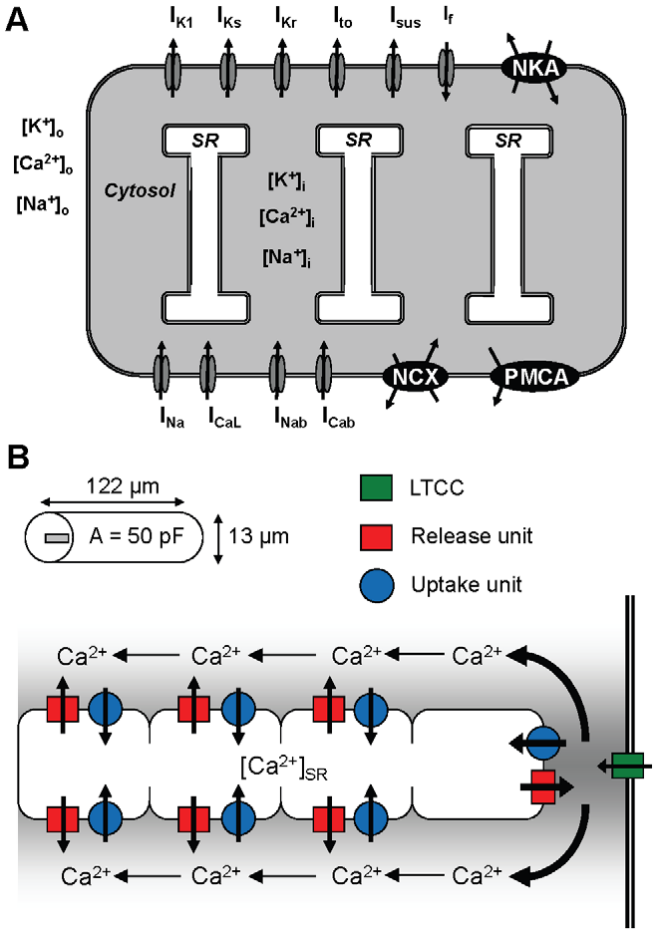
The human atrial myocyte model. (A) Membrane currents and a schematic overview of the model. The SL currents presented by the model are the transient outward K^+^ current (*I*_to_), the slow delayed rectifier K^+^ current (*I*_Ks_), the rapid delayed rectifier K^+^ current (*I*_Kr_), the sustained outward K^+^ current (*I*_sus_), the time-independent K^+^ current (*I*_K1_), the hyperpolarization-activated inward K^+^ current (*I*_f_), the fast Na^+^ current (*I*_Na_), the L-type Ca^2+^ current (*I*_CaL_), the PMCA, NKA, NCX, and the Na^+^ and Ca^2+^ background currents (*I*_Nab_ and *I*_Cab_). (B) The geometry and SR structure of the model. The intracellular cytosol is divided into the junctional and bulk cytosols. The former is a 0.02 µm deep region below the cell membrane, and the latter represents the rest of the cytosol below the junctional cytosol. The bulk cytosol and the SR are divided into four compartments that are 1.625 µm deep; note: each red entity contains RyR, SERCA, and SR leak. The RyR and SERCA in the first SR compartment interact with the LTCC in the junctional cytosol compartment, whereas the RyRs and SERCAs in the other three SR compartments interact with the corresponding cytosolic compartments. This scheme replicates the structure seen in immunolabeleled images of RyRs and SERCAs in atrial myocytes, in which the non-junctional release sites form a regular structure with ∼2 µm distances and the junctional release site is apart from this structure [12], [13].

The dynamic components of the model cell are the membrane current-voltage system and the intracellular Ca^2+^, Na^+^, and K^+^ and SR Ca^2+^ concentrations (Figure 1). The intracellular and SR Ca^2+^ also have a spatial dimension. The cell was modeled as a cylinder, with a length of 122.051 µm and a radius of 6.02 µm (Figure 1B). These yield a 50 pF capacitance for the cell membrane when the top and bottom of the cylinder are not included in the area [10]. The intracellular space is divided into the junctional cytosol, which is a 0.02 µm deep region below the cell membrane [11], [12], and the bulk cytosol, which represents the rest of the cytosol below the junctional cytosol. The bulk cytosol and the SR are further divided into four 1.625 µm deep compartments (Figure 1B). The RyR and SERCA in the first SR compartment interact with the junctional cytosol compartment, whereas the RyRs and SERCAs in the other three SR compartments interact with the corresponding bulk cytosolic compartments (Figure 1B). This structure in our model is similar to the structure seen in immunolabeleled images of RyRs and SERCAs in atrial myocytes, in which the non-junctional release sites form a regular structure with ∼2 µm distances in the transverse direction and the junctional release site is apart from this structure [12], [13]. The volume of the SR was set to 2.25% of the volume of the bulk cytosol in each of these compartments, and thus also in the whole cell [14]. The complete set of geometrical and physical parameters is shown Table 1.

**Table 1:** Cell geometry and physical parameters.

Parameter	Definition	Value	Value [ref.]
*F*	Faraday constant	96478 C/mol	
*R*	Ideal gas constant	8314 mJ/(mol K)	
*T*	Temperature	306.15 K	
[Na^+^]_o_	Extracellular Na^+^ concentration	130 mM	
[Ca^2+^]_o_	Extracellular Ca^2+^ concentration	1.8 mM	
[K^+^]_o_	Extracellular K^+^ concentration	5.4 mM	
*C_m_*	Cell membrane capacitance	0.05 nF	0.0519±0.035 nF [10]
			0.041 nF [11]
			0.058±0.078 nF [16]
*r_junct_*	Radius of the bulk cytosol	6.5 µm	4.6±0.3 µm [74]
			7.8 µm [11]
			7.4±0.15 µm [70]
*l_cell_*	Length of the cell	122.051 µm	88±6.1 µm [74]
			111.3±6.5 µm [75]
			101.1±1.5 µm [70]
*V_ss_*	Volume of the subspace	4.99232×10^−5^ nL	
*Δr*	Width of bulk cytosol compartments	1.625 µm	∼2 µm [12], [13]

### Intracellular Ca^2+^ diffusion and buffering

The Ca^2+^ diffusion in the bulk cytosol and in the SR was modeled with Fick's second law of diffusion. The components of cytosolic Ca^2+^ buffering are shown in Table 2. The effect of the mobility of Ca^2+^ buffers in the bulk cytosol was implemented as described previously [15]. The amount of SR Ca^2+^ buffer (calsequestrin) was fitted based on experimental SR Ca^2+^ content [16]; see Results for details. The diffusion between the junctional and bulk cytosol was modeled as an analytical diffusion equation [17]. The accessible volume for the Ca^2+^ diffusion in the cytosol and in the SR was set to 50% of the total volume of the compartments [11]. Also the accessible area for Ca^2+^ diffusion between junctional and bulk cytosol was set to 50% of the total area between these compartments.

**Table 2:** Parameters of the Ca^2+^ buffering.

Parameter	Definition	Value [ref]
*D_Ca_*	diffusion coefficient for Ca^2+^	780 µm/s [76]
*D_CaBm_*	diffusion coefficient for Ca^2+^-buffer complex	25 µm/s [11]
*D_CaSR_*	diffusion coefficient for Ca^2+^ in SR	44 µm/s [18], [77]
[BCa]	Arbitrary cytosol Ca^2+^ buffer	0.024 mM [2]
[SLlow]	Phospholipid concentration (low-affinity sites)	165 mM [78]
[SLhigh]	Phospholipid concentration (high-affinity sites)	13 mM [78]
*K_dBCa_*	Dissociation constant for arbitrary cytosol Ca^2+^ buffer	0.00238 mM [79]
*K_dSLlow_*	Dissociation constant for low-affinity phospholipid sites	1.1 mM [80]
*K_dSLhigh_*	Dissociation constant for high-affinity phospholipid sites	0.013 mM [80]

The diffusion coefficients are shown in Table 2. Previously, it has been shown that the effective diffusion coefficient for Ca^2+^ in the SR is 8–9 µm^2^/s [18]. The effective diffusion coefficient is smaller than the free diffusion coefficient due to the Ca^2+^ buffering in the SR [15]. In our model, the free diffusion coefficient for Ca^2+^ in the SR is 44 µm^2^/s, which yields an effective coefficient of 8–12 µm^2^/s in the relevant range of [Ca^2+^]_SR_ (0.3–0.6 mM).

The components of cytosolic Ca^2+^ buffering have not been characterized from atrial myocytes in the same detail as in ventricular myocytes [1]. Previously, ventricular data has been used to model the atrial Ca^2+^ buffering [11]. However, implementation of a ventricular buffering system in our model resulted in almost non-existent Ca^2+^ transients with the experimental SR Ca^2+^ content [16] (data not shown). In our model, the ‘known’ cytosolic Ca^2+^ buffers are the sarcolemma, which is assumed to be as in ventricular myocytes based on [11], and the SERCA [19], which is fitted based on Ca^2+^ transient kinetics and SR Ca^2+^ content. The rest of the buffering, consisting most likely of troponin, calmodulin and myosin, is modeled as an single arbitrary mobile buffer, which has the mobility and *K*_d_ of calmodulin [11].

### Sarcoplasmic reticulum

The Ca^2+^ buffering to and uptake by the SERCA and the passive Ca^2+^ leak from the SR to the cytosol were modeled as previously [19]. See Table S1 in the Supporting Information for parameter values.

**Figure 2:**
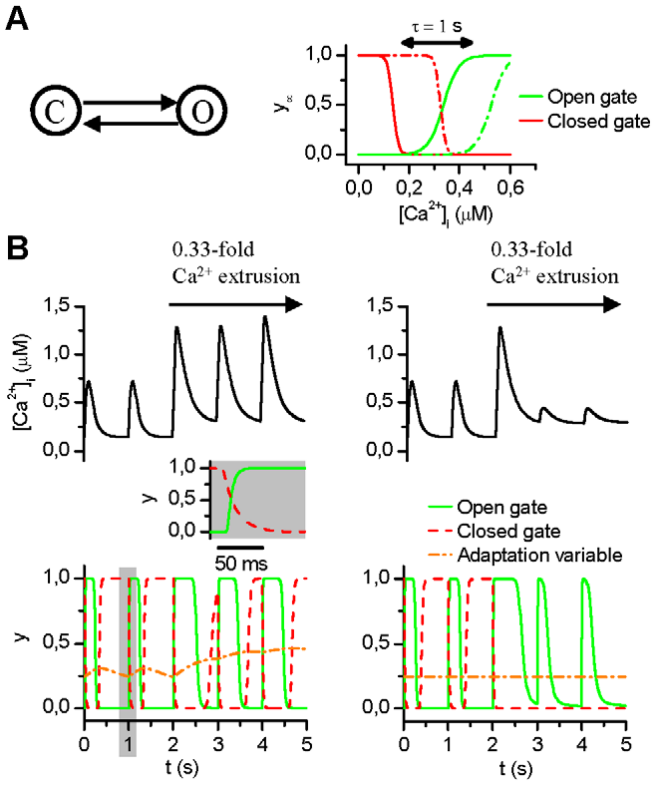
The RyR model characteristics. (A) The steady-state probabilities of open (green) and closed (red) gates are shown as a function of intracellular [Ca^2+^] for two illustrative states of adaptation. The time constant τ refers to the adaptation that modulates the steady-state probabilities of open and closed gates. (B) The effect of RyR adaptation. When the adaptation is not inhibited (left) the dependence of the RyR open probability (y axis) on the intracellular Ca^2+^ adapts in line with experimental findings [33], [34]. Inset shows the dynamics of the open and closed gate in the control situation. The 0.33-fold Ca^2+^ was implemented by reducing the maximal currents/rates of the NCX, SERCA and PMCA to 33% of control.

To describe the Ca^2+^ release flux through the RyR channel, we developed a novel phenomenological model (Figure 2) that employs a Hodgkin-Huxley type of formalism. The model has three gating variables (Figure 2A): adaptation gate, open gate and closed gate. The complete set of equations (Text S1) and the parameter values (Table S2) are listed in the Supporting Information. Time constants of RyR gating were adjusted so that the calcium release from a release unit situated in non-junctional compartments represents a signal that is spread wider both spatially and temporally compared to the junctional space (Table S2). The simple structure makes the RyR model computationally efficient, especially for modeling the Ca^2+^ wave propagation, and limits the amount of unnecessary free variables. However, as shown in Figure 2B, the model is still capable of reproducing complex features of RyR Ca^2+^ release, i.e. the dependence of the release on both the intracellular and SR [Ca^2+^] and the adaptation of the RyR open probability's dependence on intracellular Ca^2+^ [20], [21].

### Sarcolemmal ion currents

The SL ion currents were mostly formulated as in the previously published model of human atrial AP [10]. All the major modifications and novel features of the ion current submodels are described in the Text S1. Minor adjustments of parameter values are listed in Table S1.

## Results

### Sarcolemmal ion currents and action potential characteristics

As in other excitable cells, APs in atrial myocytes reflect the coordinated activation of several voltage-gated inward (depolarizing) and outward (repolarizing) ion channel currents (Figure S3 in the Supporting Information). The major depolarizing currents in the initial phase of the AP are the *I*_Na_ and *I*_CaL_, and *I*_NCX_ during the later phase of the AP (Figure S3 B&C&G). The *I*_to_ and *I*_sus_ generate large repolarizing currents in the beginning of the AP, which quickly repolarize the membrane voltage back to −30 mV after the spike (Figure S3 A&D). Following this, the repolarization is carried mostly by *I*_K1_ (Figure S3E) with very little contribution by *I*_Ks_ and *I*_Kr_ (Figure S3F). Although there is a significant amount of *I*_f_ present in human atrial myocytes [22], it does not contribute substantially to the action potential (Figure S3F), since it is activated at voltages below −80 mV (see Figure S2). The PMCA creates only a very small current (Figure S3G).

As a principal validation, we compared the characteristics of the emergent AP waveform of our model to published data during 1 Hz pacing (3; see also Discussion). The model reproduces the experimental values for the resting membrane potential (−77 mV), AP upstroke velocity (170 mV/ms), AP amplitude (119 mV), and AP duration (APD; APD_30_ = 11 ms APD_90_ = 239 ms,) at different stages of repolarization (see Table S3 in the Supporting Information for detailed comparison).

### Intracellular Ca^2+^ dynamics

**Figure 3:**
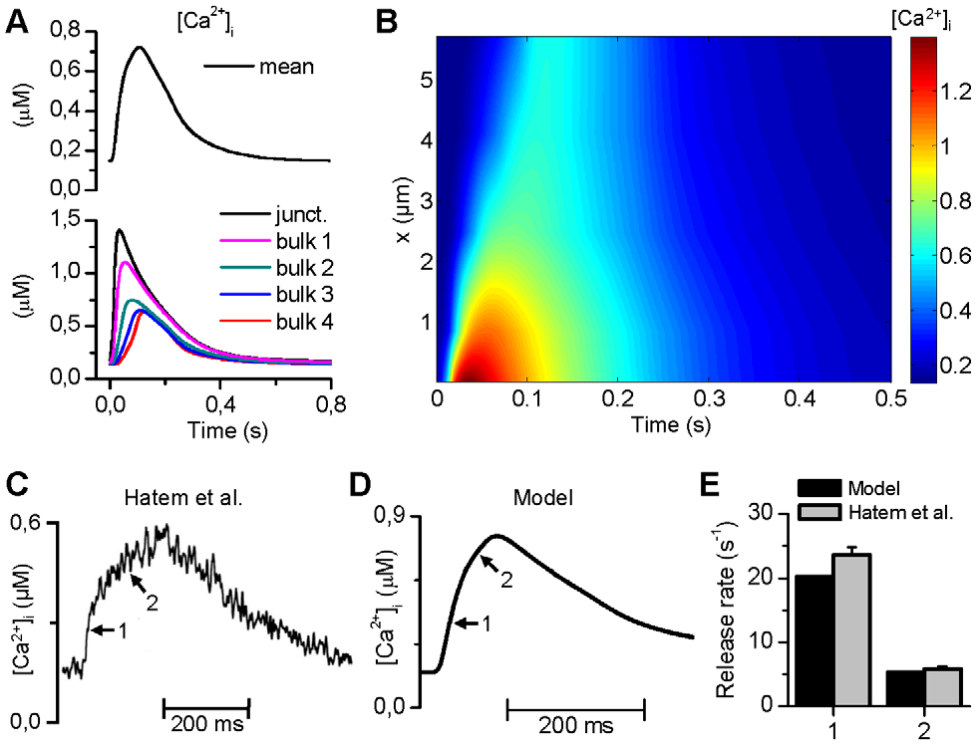
Ca^2+^ dynamics of the model. (A) The Ca^2+^ transients at different parts of the cytosol differ from the average cytosolic Ca^2+^ signal. The x-coordinates of the compartments are as follows: subspace 0.01 µm, bulk1 = 0.8325 µm, bulk2 = 2.4575 µm, bulk3 = 4.0825 µm, and bulk4 = 5.7075 µm; SL = 0 µm. (B) Spatiotemporal representation of [Ca^2+^] demonstrates clearly the divergence of both the amplitude and delay of Ca^2+^ release in different parts of the cytosol. (C) and (D) The model reproduces the experimentally found biphasic increment during the upstroke of the global Ca^2+^ transient [7]. (E) Release rates of the two phases of the Ca^2+^ transient in experiments by Hatem *et al*. [7] and simulations. The two values of release rates (arrows in (C) indicate the stages of release) were obtained with a linear fit to normalized Ca^2+^ transients.

In our model, the average cytosolic Ca^2+^ signal has a resting concentration of 0.15 µM and an amplitude of 0.58 µM (Figure 3A) at 1 Hz pacing. The reported single exponential decay constants of the Ca^2+^ transient range from 92 ms to 160 ms [4], [23], [24] and in comparative studies between atrial and ventricular myocytes, the atrial myocytes have more rapid decays [3], [4], [25]. In our model, the decay constant of the Ca^2+^ transient is 131 ms, which is in line with data from atrial myocytes and more rapid than that reported in human ventricular myocytes [26] (Table 3). As a high-level validation of the relative contribution of Ca^2+^ transport mechanisms that underlie Ca^2+^ dynamics of the model, we simulated the effect of elevation of extracellular Ca^2+^ from 0.9 to 3.2 mM (data not shown). In line with experimental findings [27], the diastolic and systolic [Ca^2+^]_i_ increased by 52% and 88%, respectively.

**Table 3:** Comparison of Ca^2+^ characteristics of the developed model and experiments.

Parameter	Simulated value	Experimental value [ref]
*Ca_diast_*	0.146 µM	0.164±0.008 µM [70] T = 23°C
		0.066±0.008 µM; rat atrial [4] T = 23°C
*Ca_syst_*	0.723 µM	0. 251±0.039 µM; rat atrial (67% of ventricular) [4] T = 23°C
Δ[*Ca^2+^*]*_i_*	0.577 µM	0. 185±0.039 µM ; rat atrial [4] T = 23°C
	peripheral peak 1.4 µM; center/peripheral ampl = 0.41	∼1 µM peripheral peak and only a small rise in center; rat atrial [28] T = 22°C
[*Ca^2+^*] *time-to-peak peripheral/center half peak*	34.8 ms (ss), 54.8 ms (1^st^ bulk)/123.6 ms (last bulk)	57.1±4.0 ms/123.7±7.8 ms; rat atrial [3]
	15.0 ms (ss), 23.1 ms (1^st^ bulk)/82.6 ms (last bulk)	6.3±0.5/71.6±16.7 ms; rat atrial [3]
*Ca^2+^ τ*	130.8 ms	shorter than ventricular cell [81]
		∼160 ms; dog [23]
*Ca 50% relax time*	122.9 ms	177.5±9.0 [27] T = 37°C
*Ca^2+^ transient duration*	∼600 ms	539.1±31.0 [27] T = 37°C
*Ca^2+^ decay rate constant (1/τ)*	7.28 1/s	7.4±0.6 1/s; rat atrial [4] T = 23°C
		10.9±0.7 1/s; cat atrial [24] T = 22–25°C
*SR Ca^2+^ content*	76.2 µM	50.9±7.4 µmol/l/cytosol [16] T = 23°C
*Ca^2+^ propagation speed*	79.0 µm/s↔12.7 ms/µm (estimated from contour plot)	91.2±12.2 µm/s; rat atrial [3]
		10.1±2.7 ms/µm [82]
	peripheral peak at 34.8 ms	∼20 ms; rat atrial [28]

Although the local Ca^2+^ release homogenizes the cytosolic Ca^2+^ signal, the Ca^2+^ transients at different parts of the cytosol differ from the average signal (Figure 3A). In atrial myocytes, the [Ca^2+^]_i_ can peak in the periphery of the cell to ∼1 µM during slow pacing, whereas in the center there is only a small increase in Ca^2+^ concentration [28]. This spatial heterogeneity is reproduced by our model (Figure 3 A&B). Also the temporal characteristics of the simulated Ca^2+^ dynamics agree well with *in vitro* findings. The time-to-peak for Ca^2+^ in peripheral cytosol in atrial cells has been estimated to be ∼20 ms [28] and 57.1±4.0 ms [3], and at the center of cell 123.7±7.8 ms [3]. In the model, the peripheral peak is at 34.8 ms and the central peak at 123.6 ms, in line with the experimental data.

As shown in Figure 3 C&D, the delay between the peripheral and central Ca^2+^ release in atrial myocytes yields a biphasic increment during the upstroke of the whole cell Ca^2+^ transient [7]. This phenomenon is reproduced in our model with similar upstroke dynamics (Figure 3E) as recorded in human atrial myocytes [7]. We simulated a voltage clamp experiment with a corresponding protocol and obtained the release rates with a linear fit to normalized Ca^2+^ transients.

### SR Ca^2+^ dynamics

In human atrial myocytes, the diastolic SR Ca^2+^ content, measured as the integral of NCX current during a caffeine-induced Ca^2+^ transient, has been reported to be 8.3±1.2 amol/pF [16]. Based on the calculations of Hove-Madsen *et al*. [16], this corresponds to 50.9±7.4 µM of accessible cytosolic volume with the dimensions of our model. The SR Ca^2+^ content in our model is 76.2 µM. However, if we use the model to reproduce the caffeine-pulse experiment [16] and calculate the integral of the generated NCX current, we get a comparable value of 7.5 amol/pF for the SR Ca^2+^ content in the model (data not shown). A possible source for the difference between the integrated NCX value and the actual SR Ca^2+^ content is the fraction of Ca^2+^ that is extruded from the cell by the PMCA, which was not considered in the experimental analysis [16].

**Figure 4:**
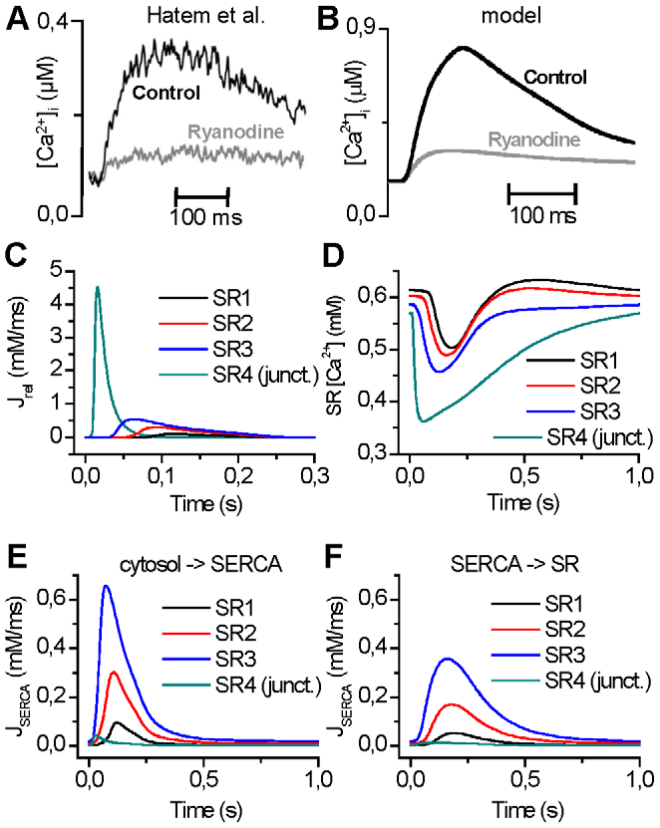
SR Ca^2+^ dynamics of the model. (A) and (B) Inhibition of the SR Ca^2+^ release with ryanodine greatly reduces the Ca^2+^ transient amplitude in both experiments [7] and simulations. Detailed analysis indicates that the SR Ca^2+^ release generates 77% of the Ca^2+^ transient amplitude, which is in line with the experimental findings 79±6% of Hatem *et al*. [7]. (C) and (D) Most of the Ca^2+^ release is generated from the junctional compartment. (E) and (F) During the uptake of Ca^2+^ from the cytosol to the SR, the SERCA buffers the Ca^2+^ and generates a delay in the fluxes between the cytosol to SERCA and SERCA to SR. At the end of the diastolic phase, there is some diffusion of Ca^2+^ in the SR, which balances the concentration differences in different parts of the SR.

The Ca^2+^ release from the SR generates 79±6% of the Ca^2+^ transient amplitude in human atrial myocytes [7] and 77% in our model (Figure 4D). Most of the Ca^2+^ release is generated in the junctional compartment (Figure 4 A&B). During the uptake of Ca^2+^ from the cytosol to the SR, the SERCA buffers the Ca^2+^ and generates a delay in the fluxes between cytosol to SERCA (Figure 4C) and SERCA to SR (Figure 4D). At the end of the diastolic phase there is some diffusion of Ca^2+^ in the SR, which balances the concentration differences in different parts of the SR (Figure 4D).

### Role of junctional and non-junctional SR Ca^2+^ release

**Figure 5:**
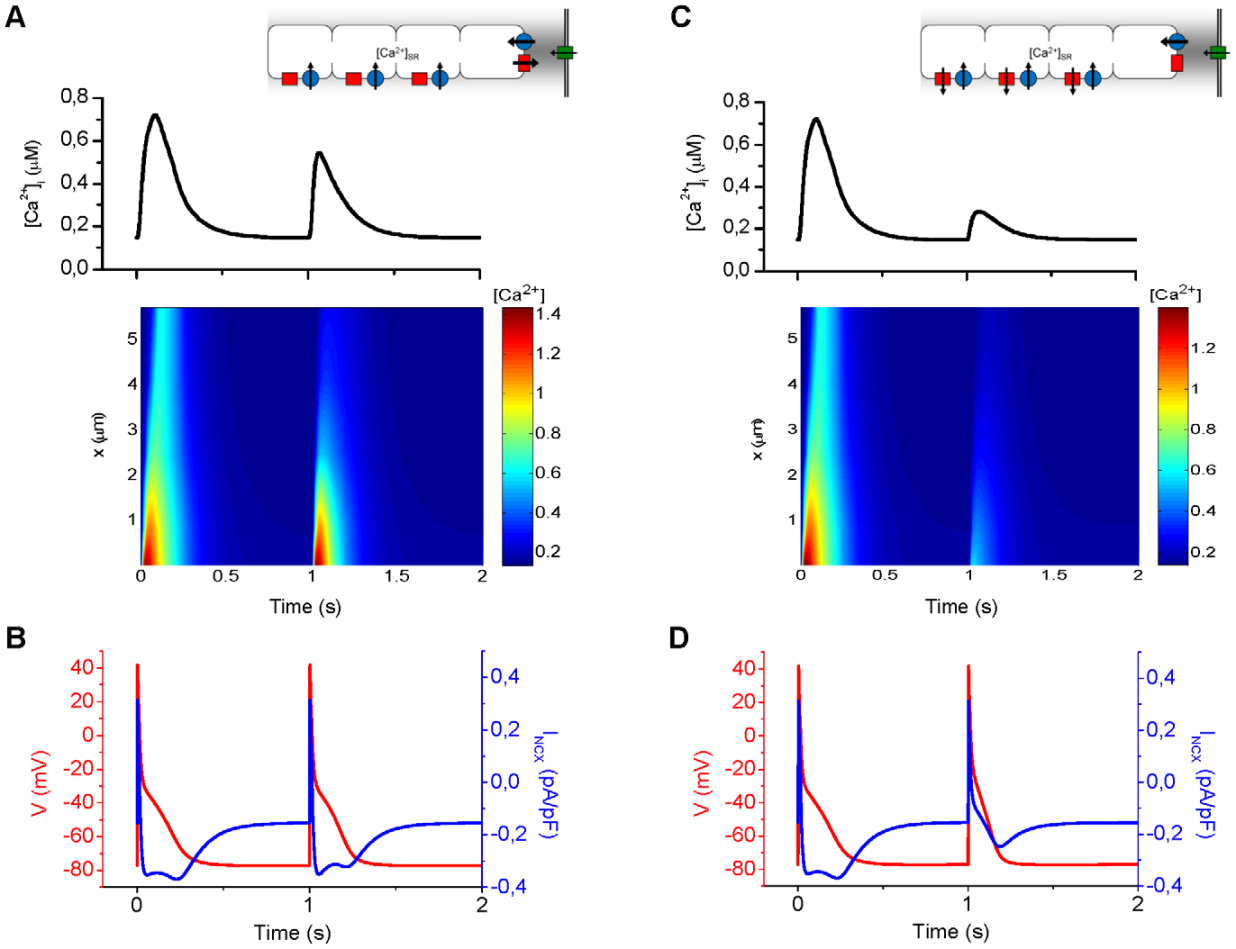
Role of the junctional vs. bulk SR Ca^2+^ release sites in the ECC. (A) Inhibition of the release sites (at t = 1 s) in the bulk cytosol has only a small impact on the ECC. The amplitude of the global Ca^2+^ transient is decreased by 31%. The Ca^2+^ signal becomes relatively inhomogeneous and it is not carried at all to the center of the cell. (B) The abnormal Ca^2+^ dynamics also affect the AP: APD_90_ is shortened by 10%. Since there is less Ca^2+^ to be extruded, the *I*_NCX_ is reduced. (C) Inhibition of the junctional SR Ca^2+^ release site (at t = 1 s) has a profound effect on the ECC. The amplitude of the global Ca^2+^ transient is decreased by 58%. Without the junctional SR Ca^2+^ release, the Ca^2+^ influx via the L-type Ca^2+^ channels is too weak to trigger the CICR at the first release site in the bulk cytosol. Thus, the inhibition of the junctional release results in a failure in the propagation of the Ca^2+^ signal. (D) The APD_90_ is shortened by 34%. Due to the lower [Ca^2+^]_i_ in the junctional compartment, the depolarizing *I*_NCX_ is not activated to the normal level.

Having established that the AP characteristics and Ca^2+^ dynamics of our model are in line with *in vitro* findings, we wanted to exploit the potential of the model to elucidate the roles of junctional and non-junctional SR Ca^2+^ release sites. Accordingly, we conducted two *in silico* experiments, in which either the non-junctional (Figure 5A) or junctional (Figure 5C) SR Ca^2+^ release was blocked. Results indicate that inhibition of the release of non-junctional sites in the bulk cytosol has only a small impact on the ECC (Figure 5A). The amplitude of the global Ca^2+^ transient is decreased by 31% and the APD at 90% repolarization (APD_90_) is decreased by 10% (Figure 5B). The AP appears to be shortened because the lower [Ca^2+^]_i_ in the junctional compartment does not activate the depolarizing *I*_NCX_ (Figure 5 B) to the normal extent.

The most significant effect of inhibiting the release sites in the bulk cytosol is that the Ca^2+^ signal becomes relatively inhomogeneous and it is not carried at all to the center of the cell (Figure 5A, lower panel). Thus, the release sites in the bulk cytosol are not a significant source of Ca^2+^ but act more as amplifiers of the Ca^2+^ signal during fire-diffuse-fire propagation.

Compared to the previous scenario, inhibition of the junctional SR Ca^2+^ release site yields partly opposite results (Figure 5C). The amplitude of the global Ca^2+^ transient is decreased by 58% and the APD_90_ is decreased by 34% (Figure 5D). The AP is shortened, because the [Ca^2+^]_i_ in the junctional compartment is elevated only slightly and thus the activation of the depolarizing *I*_NCX_ is reduced dramatically (Figure 5 D). However, similar to the situation where the release sites in the bulk cytosol were inhibited (Figure 5A), inhibition of the junctional release results in a failure in the propagation of the Ca^2+^ signal to the center of the cell. Without the junctional SR Ca^2+^ release, the Ca^2+^ signal coming from the LTCCs is too weak to trigger the CICR at the first release site in the bulk cytosol (Figure 5C, lower panel).

### Intracellular Ca^2+^ transient and action potential duration

**Figure 6:**
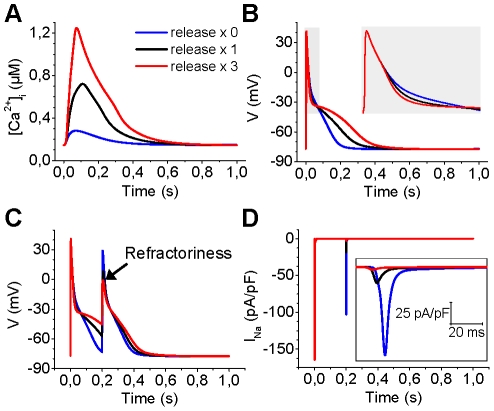
Effect of SR Ca^2+^ release on APD and refractoriness. (A) Modulation of SR Ca^2+^ release (total block, blue; normal, black; triple release, red) has a dramatic effect on the mean cytosolic Ca^2+^ transient. (B) Increased release promotes slower initial (inset: zoomed to the first 75 ms) and faster late repolarization of the membrane voltage, whereas blocking the release generates opposite changes. (C) The SR Ca^2+^ release is an important factor for the refractoriness of the cell. Increased SR Ca^2+^ release can block the second AP (interval 200 ms). Note that the second peak that is seen in the AP trace (red line) is caused by the stimulus current, i.e., there is no actual second AP. (D) Refractoriness is caused by blocking the *I*_Na_, which is shown more clearly in the inset.

Above we have shown that our myocyte model can reproduce the experimentally observed impact of SR Ca^2+^ release on the inactivation of *I*_CaL_ (Figure S1 *C*) and amplitude of the Ca^2+^ transient (Figure 4B). In addition, our results indicate that the SR Ca^2+^ release, especially junctional, affects the membrane voltage also via the NCX (Figure 5 B&D). To further study the effect of intracellular Ca^2+^ dynamics on AP morphology, we simulated the acute effect of total block and 3-fold increase of Ca^2+^ release (Figure 6). The results indicate that the amplitude of the Ca^2+^ transient (Figure 6A) has a substantial effect on the APD (Figure 6B). Blocking the SR Ca^2+^ release slows down the early phase of repolarization (Figure 6B, inset), but speeds up the late repolarization (Figure 6B), whereas the 3-fold increase of Ca^2+^ release has an opposite effect. Slowed AP repolarization causes the sodium channels to remain inactivated for a longer time. Consequently, the duration of the refractory period is increased and a prematurely applied second stimulus is unable to trigger the next AP (Figure 6D).

**Figure 7:**
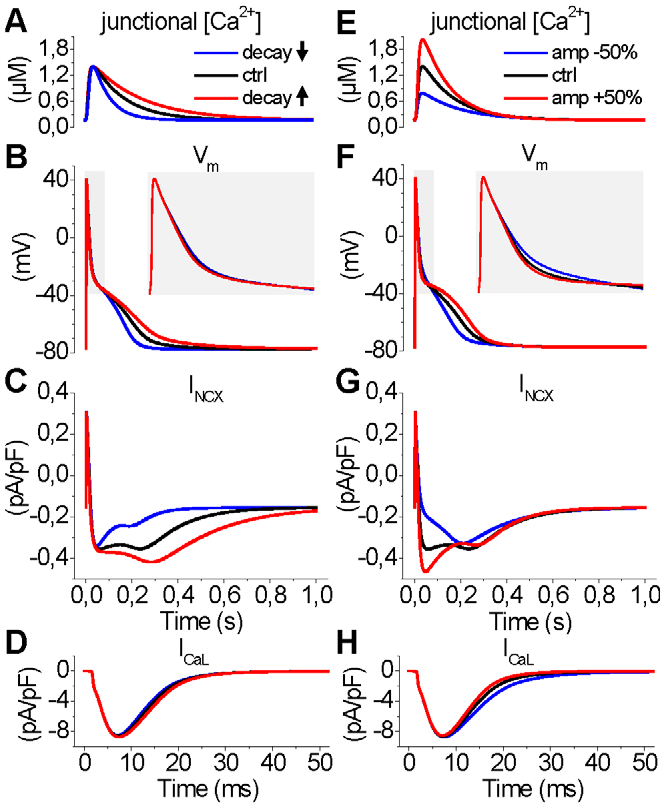
Effect of the amplitude and decay of the junctional Ca^2+^ transient on APD. (A) The junctional Ca^2+^ transient was clamped to three different decay modes (slowed decay, blue; normal decay, black; accelerated decay, red) with analytical functions. (B) Modulating the decay of the junctional Ca^2+^ transient has a pronounced effect on the APD (inset: zoomed to the first 75 ms). (C) The modulatory effect is mainly mediated by the changes in the late phase of *I*_NCX_. The late current increases/decreases with faster/slower decay of the Ca^2+^ transient, respectively. (D) The modulation of Ca^2+^ transient decay has no significant effect on the inactivation the *I*_CaL_. (E) To mimic the modulation of RyR (decreased release, blue; normal release, black; increased release, red), the junctional Ca^2+^ transient was clamped to three different amplitude modes (inset). (F) Increasing the amplitude of the junctional Ca^2+^ transient accelerates the early repolarization and decelerates the late repolarization of the AP (inset: zoomed to the first 75 ms), while a decreased amplitude has an opposite effect. G) The modulatory effect of the Ca^2+^ transient amplitude is mainly mediated by the changes in the initial phase of *I*_NCX_. The late current increases with faster and decreases with slower decay of the Ca^2+^ transient. (H) The modulation of Ca^2+^ transient amplitude also affects the inactivation the *I*_CaL_.

To further dissect the role of [Ca^2+^]_i_ in AP morphology, we simulated separately the effect of decay and amplitude modulation of intracellular Ca^2+^ transients on the APD (Figure 7). That is, the junctional [Ca^2+^] was “clamped” to three different modes (Figure 7 A&E) in both cases. This modification was implemented by replacing differential variable of junctional [Ca^2+^] with an analytical equation that was fitted manually to the simulated control [Ca^2+^] trace. Then either the decay (Figure 7A) or the amplitude (Figure 7E) of the junctional Ca^2+^ transient was modified. As the results show, the accelerated decay shortens the AP substantially, whereas deceleration of decay has an opposite effect (Figure 7B). The decay modulation has little or no effect on the early repolarization of the membrane voltage (Figure 7B, inset). The underlying mechanism appears to be the changed *I*_NCX_ (Figure 7C), whereas *I*_CaL_ is not affected (Figure 7D).

Similar to the effect of decay modulation, the amplitude of the [Ca^2+^] transient affects the APD substantially (Figure 7F). However, compared to the modulation by the [Ca^2+^]_i_ transient decay, increasing the amplitude also affects the early phases of repolarization (Figure 7F, inset) by enhancing the outward peak current via the NCX and accelerating the inactivation of *I*_CaL_ (Figure 7H). This results in a more complex modulation scheme, in which the increased [Ca^2+^] transient amplitude accelerates the early repolarization and decelerates the late repolarization (Figure 7F).

### Rate dependence of action potential duration

Increasing the pacing rate causes an immediate (within a few APs) and then a gradual (reaching steady state over several minutes) decrease in the APD of atrial myocytes; this has been shown in numerous studies. Experimental findings indicate that this adaptation coincides with functional changes in the LTCC following calcium overload [29], [30], and it is thus seen as one of the main mechanisms that underlie the changes in the ADP [31], [32]. However, in ventricular myocytes, one of the important factors in rate dependence has been shown to be the accumulation of cytosolic Na^+^ during fast pacing [33], [34], [35], [36]. To further study this phenomenon in atrial myocytes, we simulated pacing experiments within a physiologically relevant range of frequencies or basic cycle lengths (BCLs).

**Figure 8:**
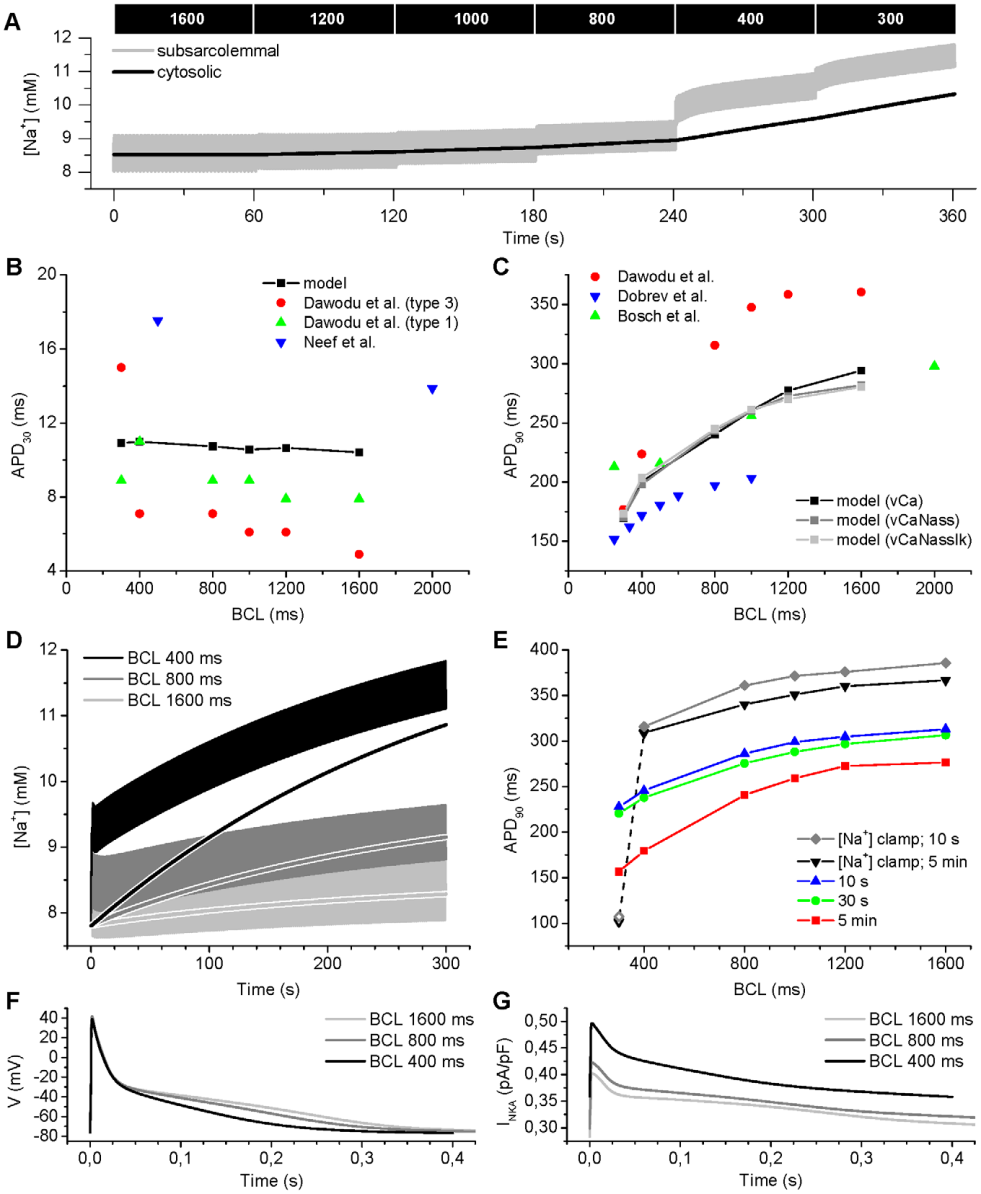
Role of Na^+^ accumulation in the rate dependence of the AP. The myocyte was paced as previously [38] with a stepwise reduction in BCL, and corresponding AP characteristics were defined for each BCL. (A) Shortening of the BCL increases both the subsarcolemmal and cytosolic [Na^+^], especially at BCLs 400 ms and 300 ms. (B) The values of APD_30_ calculated from simulations are compared to those reported by Dawodu *et al*. [38], and Neef *et al.* [70]. (C) The steep dependence of APD_90_ on the BCL reported by Dawodu *et al*. [38], Bosch *et al*. [72] and Dobrev & Ravens [73] is reproduced by the model. (D) Continued fast pacing starting from a quiescent steady-state results in a dramatic accumulation of intracellular Na^+^. (E) APD adaptation in control situation conditions and [Na^+^]_i_ “clamped” to the quiescent steady-state value. (F) AP shape at BCLs 1600, 800 and 400 ms after 5 minutes of pacing starting from a quiescent steady-state. (G) Accumulation of Na^+^ due to fast pacing, continued for 5 minutes, increases Na^+^/K^+^-ATPase current (*I*_NKA_) substantially.

To account for the other rate-dependent mechanisms that affect the APD, we implemented two additional variants of the myocyte that are described in detail in the Supporting Information (Text S1). Briefly, we added a subsarcolemmal Na^+^ compartment (model variant: “vCaNass”) to the developed model (vCa), and we also included the recently updated description of K^+^ currents according to [37] (vCaNassIk). Simulation results shown in Figure 8 were obtained as in a previously used experimental protocol [38]. Figure 8A shows the overall changes in [Ca^2+^]_i_ dynamics: diastolic and systolic [Ca^2+^]_i_ increase and decrease with faster pacing, respectively, and accumulation of intracellular Na^+^. The simulated values of APD_30_ that were calculated for each BCL fall within the range of reported experimental values (Figure 8B). The steep dependence of APD_90_ on the BCL is reproduced faithfully by the model (Figure 8C). Although the variance of absolute APD_90_ values reported in the literature is large, the shape of the curve is similar in experiments and simulations. The relative change of APD_90_ in the range of BCL = [1600, 400] in our model (−32%, −30% and −27% for the model variants vCa, vCaNass and vCaNassIk, respectively) fits well to the *in vitro* values of Boutjdir *et al*. [39] (−44%) and Dawodu *et al*. [38] (−38%).

A multitude of measurement protocols are used to study the rate dependence of AP morphology in cardiac myocytes. Most importantly, the length of the period, after which the APD is determined, ranges from tens of seconds [31], [40] to five minutes [41]. As the continued pacing potentially increases the [Na^+^]_i_, we wanted to study the rate dependence of AP with a second pacing protocol, in which simulation is always started from the quiescent steady-state and continued for five minutes for each BCL, separately (representative data is shown in Figure 8D). To account for all rate-dependent mechanisms described above, we performed these simulations with the vCaNassIk model variant.

Comparison of APD_90_ after 30 seconds and 5 minutes of pacing highlights the dynamic nature of rate dependence (Figure 8E). That is, the shortening of the AP in response to faster pacing becomes more pronounced as pacing is continued. This is affected substantially by the increasing [Na^+^]_ss_ (Figure 8D). If the intracellular [Na^+^] is “clamped” to the quiescent steady-state value (7.8 mM), the effect of continued fast pacing on the APD is dramatically reduced (Figure 8E, black line). Furthermore, the simulation results suggest that Na^+^ accumulation is actually a mechanism that is required for rate-dependent adaptation of APD, because the myocyte model failed to produce a normal AP at BCL 300 ms when [Na^+^] was “clamped” the quiescent steady-state value (Figure 8E, open triangle in the black trace). This failure was present already during the first few seconds of the simulation at BCL 300 ms (open diamond, in the grey trace).

Results shown in Figure 8E suggest that roughly half of the rate-dependent adaptation of APD comes from the short-term ionic mechanisms that operate in the timescale of seconds, whereas the long-term adaptation responsible for the other half of APD decrease takes minutes to develop. Furthermore, the longer pacing protocol results in a much steeper dependence of APD in the BCL range of [1600, 400] ms (−35%), compared to the shortening of APD (−27%) shown in Figure 8C.

Simulation results suggest that the mechanism that links AP shortening (Figure 8F) to Na^+^ accumulation is the Na^+^/K^+^-ATPase (NKA). The enhanced pumping function of NKA lead to a dramatic increase in the current (*I*_NKA_) (Figure 8G). Accordingly, this mechanism has been reported previously in human atrial fibers [42] and guinea pig ventricular myocytes [43]. To confirm that this feature of the myocyte model does not depend on the Na^+^ parameters of model variant, we simulated the same protocol with model variant vCa (no junctional Na^+^ compartment) and found that fast pacing resulted in a similar increase in *I*_NKA_ (data not shown).

### Rate-dependent changes in the NCX current

**Figure 9:**
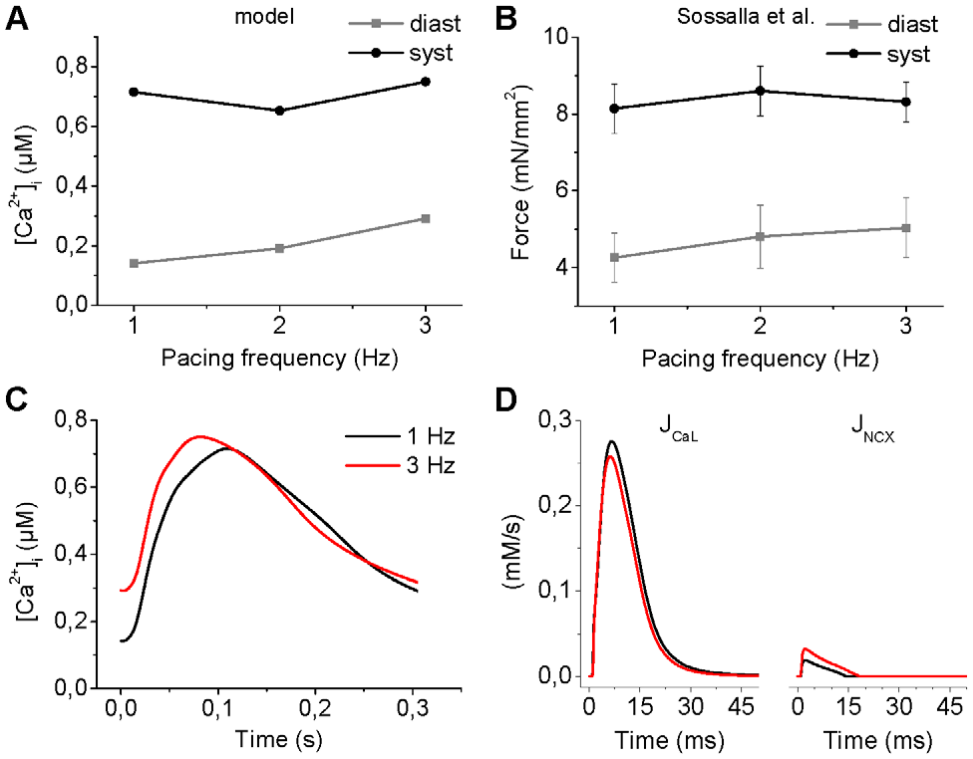
Rate dependence of Ca^2+^ dynamics. The myocyte was paced as previously [44] with a pacing frequencies [1], [2], [3] Hz. Each pacing frequency was applied for 5 minutes in a continuous stimulus train. Rate dependence of Ca^2+^ dynamics (A) matches qualitatively to the force-frequency relation (B) reported by Sossalla et al. [44]. (C) Changes of Ca^2+^ transient dynamics in response to fast pacing. (D) Ca^2+^ influx via the reverse mode of the NCX (right panel) increases substantially during fast pacing, whereas the influx via the LTCC is decreased slightly (left panel).

To evaluate the effect that Na^+^ accumulation due to fast pacing has on the Ca^2+^ dynamics via modulation of the NCX function, we simulated a previously used pacing protocol [44], in which the pacing frequency was increased in a stepwise manner from 1 to 2 Hz and further to 3 Hz. The results provide a general level validation for the rate dependence of Ca^2+^ dynamics of the model (Figure 9A) that correspond qualitatively to the measured force (Figure 9B).

To further study the role of NCX in the rate dependence of Ca^2+^ dynamics, we evaluated the role of the NCX as a secondary trigger of the CICR process. Figure 9E shows that whereas the Ca^2+^ influx via LTCC is decreased slightly (integral decreases by ∼14%) during fast pacing, the flux via reverse mode of NCX is increased considerably (integral increases over two-fold). However, since relative contribution of NCX is much smaller, we performed an additional simulation analysis to quantitate its role in rate-dependent modulation of Ca^2+^ dynamics. In line with a previous study [45], blocking the reverse mode of the NCX delays the peak of [Ca^2+^]_i_ by 1.8 ms (109.1 ms vs. 110.9 ms for control and block, respectively) when the pacing frequency is 1 Hz. This effect is more pronounced during fast pacing: the delay increases to 2.4 and 3.5 ms with 2 and 3 Hz, respectively. Thus, the results suggest that the rate-dependent modulation of NCX could mediate a shortening of the delay between the electrical excitation and the peak of Ca^2+^ transient.

## Discussion

We have developed a mathematical model of the human atrial myocyte with a spatially detailed and atria-specific description of intracellular [Ca^2+^]_i_ dynamics. The presented results indicate that the model accurately replicates crucial experimental findings: instead of a homogenous Ca^2+^ release, ECC is driven by a Ca^2+^ wave that arises first in the periphery and then propagates to the cell center [3], [7], [8]. This novel model enabled a dissective analysis of the interrelations between [Ca^2+^]_i_, sarcolemmal ion currents, and SR Ca^2+^ release. The results highlight the importance of the junctional SR Ca^2+^ release sites in defining the interface between Ca^2+^ and AP, whereas the non-junctional release sites are significant for the fire-diffuse-fire propagation of Ca^2+^ diffusion. Furthermore, our analysis indicates that intracellular Ca^2+^ dynamics are strongly linked to the AP morphology via modulation of NCX current, thus also affecting the refractoriness and excitability. The presented simulations also suggest that one of the main mechanisms of AP rate dependence is the accumulation of [Na^+^]_i_ that modulates the function of NKA during continued fast pacing.

### Principal characteristics of the model and relevance to previous *in silico* studies

It is well known that in the human atria the cellular AP shape varies depending, for example, on gender, age and personal ongoing medication plan. In addition to the physiological variation, studies using human tissue samples are always affected by the parallel pathophysiology of the patients who are undergoing surgery due to some other cardiac malfunction. Experimentally, the morphology of the human atrial AP ranges from a triangular AP shape with no sustained plateau to a long AP with a spike-and-dome shape [46], [47], [48]. The AP heterogeneity has been shown to correlate tightly with the relative expression levels of ion channels in different regions of the atria in an experimental canine model [49] and in human patients [50]. In the model presented here, the AP characteristics (resting membrane potential, upstroke velocity, amplitude and duration) emerging from an accurate description of individual ion currents are well within the range of experimental data reported in the literature.

During the last decade, mathematical modeling has become an established complement to experimental work in attempts to elucidate the ionic mechanisms that underlie the electrophysiology of cardiac myocytes [9]. In the case of human atrial myocyte models, the platform was established by the individual works of Nygren *et al*. [10] and Courtemanche *et al*. [31]. The usability of these comprehensive frameworks has been established, e.g. in consecutive *in silico* studies of AP morphology [37], [51] and atrial fibrillation [52], [53], [54]. While these models provide a detailed description of the transmembrane ion currents, very little emphasis has been placed on the accurate description of the spatiotemporal properties of intracellular Ca^2+^. Experimental findings indicate that there are substantial spatiotemporal differences in the properties of the atrial and ventricular Ca^2+^ transients [3], [4], thus we have developed a novel model that considers the atrial-specific properties of Ca^2+^ signaling.

### Ca^2+^ dynamics of the model

In contrast to the virtually uniform Ca^2+^ release in ventricular myocytes [6], it is characteristic for atrial myocytes that the Ca^2+^ wave, initiated by the CICR mechanism, arises first in the periphery and then propagates to the cell center [3], [7], [8]. Consequently, the delay between the peripheral and central Ca^2+^ release in atrial myocytes yields a biphasic increment during the upstroke of the Ca^2+^ transient [7]; a phenomenon that is reproduced in our model with accurate spatiotemporal parameters. This delay can be decreased by inotropic interventions that promote increased SR Ca^2+^ content, and consequently enhanced SR Ca^2+^ release; thus, it establishes a mechanism through which the interval between the electrical excitation and the peak of Ca^2+^ transient can be modulated.

Our results highlight the crucial role of the junctional SR in mediating the CICR, while the inhibition of non-junctional Ca^2+^ release sites causes only an attenuation of the Ca^2+^ signal during fire-diffuse-fire propagation. Furthermore, junctional SR Ca^2+^ release sites appear to define the interface between Ca^2+^ and AP, i.e., decreasing the junctional Ca^2+^ release shortens the AP substantially. These findings are in line with the previously suggested role of the non-junctional SR as an inotropic release reserve that can be recruited when greater contractility is required [13].

Cumulative evidence suggests that changes in the [Ca^2+^]_i_ homeostasis may initiate electrical remodeling during atrial fibrillation, which is characterized by a marked shortening of the action potential plateau phase [55], [56], [57]. In future studies, the presented model, which is based on an atrial-specific description of Ca^2+^ signaling, has thus great potential in elucidating the function of the remodeled cells with altered Ca^2+^ homeostasis and SL ion currents.

### Impact of Ca^2+^ transient on AP morphology and cellular excitability

The tight coupling of SL ion currents, which underlie the AP shape, and the [Ca^2+^]_i_ has been well established in experimental setups of both ventricular [33], [58], [59] and atrial myocytes [48], as well as in computational studies [36], [60]. Our results suggest that both amplitude and decay modulation of the Ca^2+^ transient produce significant changes to the APD compared to the control situation. Changes in [Ca^2+^]_i_ in the vicinity of the SL affects both *I*_NCX_ and *I*_CaL_. However, while both increased amplitude and decelerated decay of the Ca^2+^ transient enhance the inward *I*_NCX_, the inactivation of *I*_CaL_ is affected only by the former. These findings indicate that in human atrial myocytes the NCX is more important than the LTCC in linking the amount of Ca^2+^ released from the SR to changes in the APD. This scheme also concurs with a previously reported role of NCX as the main mediator of the inotropic effect of AP prolongation in canine atrial myocytes [61]. Understanding the role of this mechanism is of great importance because it not only links inotropic interventions (increased amplitude of the Ca^2+^ transient) but also situations such as hypothyroidism (slowed decay of the Ca^2+^ transient) to APD modulations.

When the effects of amplitude and decay modulation of the Ca^2+^ transient are combined to a strongly modulated physiological frameset of blocked or increased SR Ca^2+^ release, the resulting changes in AP morphology are even more pronounced. That is, decreasing the release decelerates the early repolarization and accelerates the late repolarization of the membrane voltage, whereas increased SR Ca^2+^ release promotes a faster early repolarization and a slower late repolarization of the AP. Most interestingly, the increased SR Ca^2+^ release enhances the refractoriness of the AP. That is, the decelerated repolarization of the AP (due to increased *I*_NCX_) slows down the recovery from inactivation of the sodium current and thus a premature electrical stimulus is unable to trigger a second AP.

### Rate-dependent modulation of action potential

The primary physiological context, in which the duration of the AP is modulated, relates to changes in heart rate [62]. Numerous parameters of the *in situ* heart are affected simultaneously in that dynamic process, but in single cell preparations the situation is significantly simpler. In ventricular myocytes, one of the main mechanisms that has been shown to underlie the rate dependence of the AP is the accumulation of cytosolic Na^+^ during fast pacing [33], [34], [35], [36]. That is, as the pacing rate increases, the Na^+^ influx per unit time is increased. Since this change is not fully compensated with increased efflux, [Na^+^]_i_ increases with continued fast pacing.

As previously demonstrated in ventricular myocytes [35], this mechanism emerges from the interplay of NKA and NCX. If only NKA or NCX senses the accumulated [Na^+^]_i_ in response to continued fast pacing, the APD is decreased less compared to the normal situation, in which both of them sense an increased [Na^+^]_i_. A similar phenomenon is apparent in our atrial model: the mere increase in pacing frequency (or reduction in BCL) does not produce a maximal shortening of the AP. Rather, the increase of [Na^+^]_i_ and the consequent change in the NKA function, caused by the continued high frequency pacing, promote a substantial further decrease of APD. Furthermore, the results indicate that the other ionic determinants of APD, emerging from the rate dependence of *I*_sus_ [37] and Ca^2+^ dynamics, are effective already within a shorter time-scale (∼10 s) of adaptation compared to that of Na^+^ (∼minutes). Thus, our findings underline the importance of considering the effect of Na^+^ accumulation in both *in vitro* and *in silico* studies of AP rate dependence when results obtained with different protocols are interpreted or compared.

In myocytes with long AP, the increase of Ca^2+^ transient shortens the AP (via inactivation *I*_CaL_). Interestingly, our simulation results indicate that, in this respect, the human atrial myocytes behave similar to animal with short AP. That is, increased Ca^2+^ transient amplitude (due to e.g. enhanced SR Ca^2+^ release) promotes an inward *I*_NCX_, thus lengthening the AP.

### Ca^2+^ entry through the NCX and its role in CICR

The role of the NCX as a trigger of SR Ca^2+^ release has been studied extensively in ventricular myocytes during recent years; the findings, however, are controversial [60]. While some results indicate that Ca^2+^ entry via the reverse mode of NCX is significant in physiological conditions [63], [64], other studies suggest it to be important only in pathological situations [1], [65].

Our simulation results suggest that in atrial myocytes the contribution of NCX to the Ca^2+^ influx is increasingly significant at higher pacing rates, even though the bulk of the flux goes via the LTCC. However, when the temporal role of these two mechanisms is compared, it is apparent that during the first few milliseconds of the CICR process the NCX contributes rather equally to the Ca^2+^ influx. Accordingly, the acute inhibition of the reverse mode of NCX delays the peak of [Ca^2+^]_i_ by a few milliseconds, depending on the pacing rate. These findings are in line with the reported delay of the Ca^2+^ transient if the Ca^2+^ entry via the NCX is inhibited in ventricular myocytes [45]. Our results thus indicate that the NCX could have an important role in accelerating the rise of [Ca^2+^]_i_ with increasing heart rate, even though LTCC is the primary trigger of CICR, as suggested previously [66]. Hence, the modulation of the NCX could promote a rate-dependent reduction of the electro-mechanical interval in the development of contractile force. The *in vivo* significance of the delay modulation by the NCX presents an interesting question for future simulation studies in tissue and/or whole-atria models.

### Potential limitations

Variability of experimental results imposes a fundamental challenge for modeling studies that utilize data measured from isolated human atrial tissues or cells. It would thus be an irrelevant and futile effort to try to fit the electrophysiological characteristics of the model perfectly to one single set of *in vitro* data. Instead, it is more essential that the simulation results agree qualitatively and semiquantitatively with the majority of the measured results, which has been established for the AP morphology in our model. There is a further challenge for validation of the Ca^2+^ dynamics due to incomplete experimental data. To compensate for this, we have also compared the model behavior to ventricular data and measurements obtained with atrial animal models.

The developed RyR module of the myocyte model is an approximate description of macroscopic SR Ca^2+^ release, sharing the general limitations ‘common pool’ models. Thus, the justification for most of the chosen parameter values cannot be derived directly from the biophysical properties of RyR channels [67], [68]. Instead, the *ad hoc* parameter values of the RyR modules are based on indirect fitting: the dynamics of intracellular Ca^2+^ were adjusted to be in line with macroscopic experimental observations [3], [7], [8]. The simple structure makes the RyR module computationally efficient, while still being capable of reproducing essential features of RyR Ca^2+^ release characterize the emergent properties of atrial Ca^2+^ dynamics: biphasity of the increasing Ca^2+^ concentration during the Ca^2+^ transient, and the relative contribution of SL and SR Ca^2+^ fluxes to the Ca^2+^ transient. A more complex RyR module might be needed in future studies if the simulations are run beyond the conditions that were investigated in this study.

Although our model can reproduce rather accurately the rate-dependent changes in ion dynamics, it should be noted that the description of the underlying mechanisms is by no means exhaustive. For example, one potential regulatory pathway is the Ca^2+^/calmodulin-dependent protein kinase (CaMK)II that has been studied extensively in ventricular myocytes [69]. Recent findings indicate that CaMKII could, for example, have an important role in the regulation of RyR [70] in atrial myocytes. As more experimental data become available, the effects of CaMKII on its phosphorylation targets should be considered in future modeling studies.

We have chosen to use Hodgkin-Huxley formalism for the ion currents. We acknowledge that Markovian models would allow for a more detailed description of the complex kinetics of processes (activation, deactivation, inactivation, and recovery from inactivation) that the channels exhibit. However, it can be very difficult to meet the information requirements for defining the transitional rate constants [71]. Furthermore, Markovian models can be computationally expensive compared to Hodgkin-Huxley formalism [71].

### Conclusion

We conclude that the novel myocyte model provides significant insight into the excitation and ion dynamics of human atria. Our results underline the tight coupling of AP morphology to SR Ca^2+^ release and intracellular Na^+^ that are subject to strong modulation under physiological conditions. Furthermore, with a physiologically accurate description of intracellular Ca^2+^ dynamics the model is a potential tool, for example, in the elucidation of mechanisms that link changes in Ca^2+^ homeostasis to pathophysiological conditions. Thus, it offers attractive possibilities to study the electrical remodeling during atrial fibrillation, and to find potential targets that affect the refractoriness of the AP. Finally, as the computational cost of the model is low, it is also a feasible component for multi-scale models of tissue and/or heart.

## Supporting Information

Figure S1 The L-type Ca^2+^ current (I_CaL_) characteristics. (A) The time-constant for gate f_1_ was fitted to experimental data [1]. In contrast to the original formulation of Nygren et al. [2] (black line), the time-constant was adjusted to have larger values in the membrane voltage range of −40 and −10 mV (blue line); an approach that has been used previously [3] (red line). (B) The steady-state curve for the Ca^2+^ dependent inactivation gate is shown as a function of [Ca^2+^] in the junctional subspace. As the lower panel shows, this formulation reproduces qualitatively the result that blocking of SR Ca^2+^ release decreases the rate of LTCC inactivation significantly in human atrial myocytes [5]. (C) The traces show the I_CaL_ that was recorded in voltage clamp simulation; holding potential = −80mV.
(0.14 MB TIF)

Figure S2 Steady-state activation and time-constant of I_f_. Experimental conditions were replicated from [12]. The current traces of I_f_ were determined by application of hyperpolarizing voltage steps from −40 to −140 mV in 10 mV steps, the holding potential was −40 mV. The I_f_ model was fitted to the left atrium wall data from [12].
(0.07 MB TIF)

Figure S3 The principal outputs of the myocyte model at 1 Hz pacing. (A) The model reproduces an AP that is characteristic for human atrial myocytes: a large initial peak with a narrow early plateau is followed by a late, low amplitude, plateau phase; a so-called triangular shape. (B) and (C) The major depolarizing currents in the initial phase of the AP are the I_Na_ and I_CaL_. (D) The I_to_ (black line) and I_sus_ (grey line) generate large repolarizing currents in the beginning of the AP. (E) The late repolarization is carried mostly by I_K1_ (F). As demonstrated by the time courses of I_f_ (black solid line), I_Ks_ (black dashed line), and I_Kr_ (grey line), they contribute very little to the AP, compared to I_to_ and I_sus_. (G) During the late phase of the AP, the I_NCX_ (grey line) generates a significant depolarizing current, while the amplitude of I_PMCA_ (black line) is much smaller.
(0.17 MB TIF)

Table S1 Modified parameter values of the previously published model components.
(0.01 MB PDF)

Table S2 Parameters of the novel RyR model.
(0.01 MB PDF)

Table S3 Comparison of AP characteristics of the developed model (vCa) and the two extended variants (vCaNass and vCaNassIk) to experiments.
(0.01 MB PDF)

Table S4 Initial values for the differential variables at 1 Hz pacing steady-state.
(0.01 MB PDF)

Text S1 Model implementation.
(0.16 MB PDF)
