# Supplementary figures and images for: Impact of Sarcoplasmic Reticulum Calcium Release on Calcium Dynamics and Action Potential Morphology in Human Atrial Myocytes: A Computational Study
Source: PLoS Comput Biol. 2011 Jan 27;7(1):e1001067. doi: 10.1371/journal.pcbi.1001067 (PMC3029229; doi:10.1371/journal.pcbi.1001067)

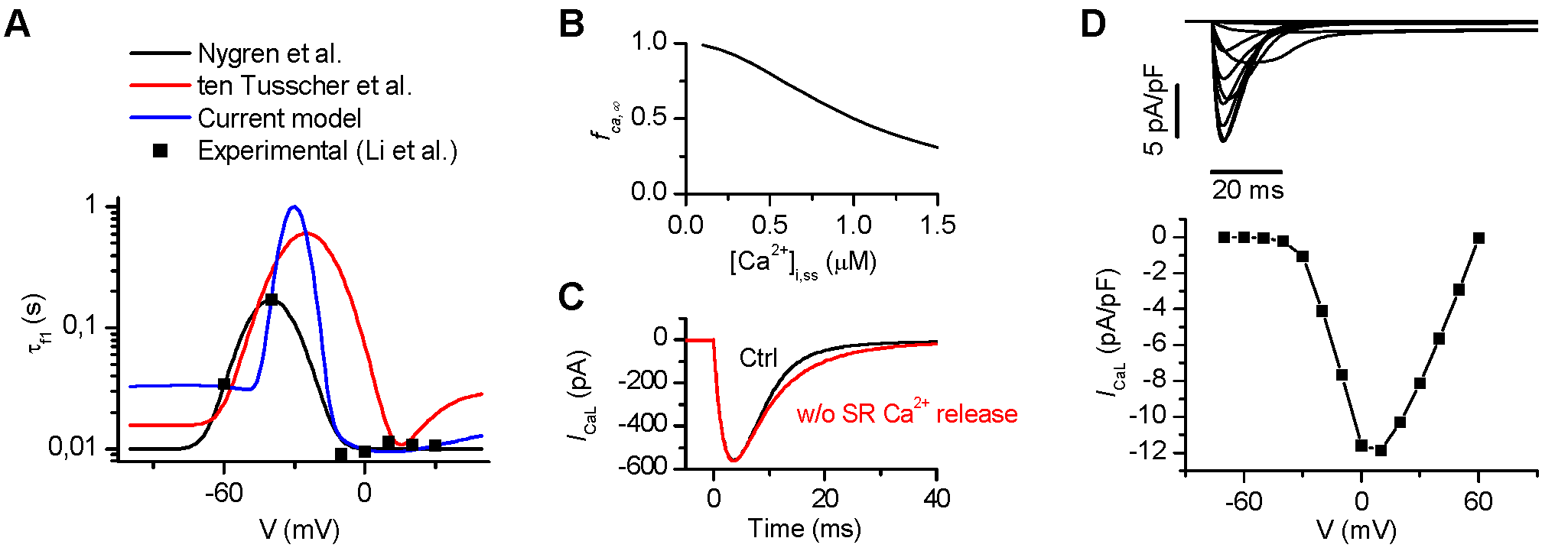

Supplement: Figure S1 — The L-type Ca2+ current (ICaL) characteristics. (A) The time-constant for gate f1 was fitted to experimental data [1]. In contrast to the original formulation of Nygren et al. [2] (black line), the time-constant was adjusted to have larger values in the membrane voltage range of −40 and −10 mV (blue line); an approach that has been used previously [3] (red line). (B) The steady-state curve for the Ca2+ dependent inactivation gate is shown as a function of [Ca2+] in the junctional subspace. As the lower panel shows, this formulation reproduces qualitatively the result that blocking of SR Ca2+ release decreases the rate of LTCC inactivation significantly in human atrial myocytes [5]. (C) The traces show the ICaL that was recorded in voltage clamp simulation; holding potential = −80mV. (0.14 MB TIF) [file pcbi.1001067.s001.tif]

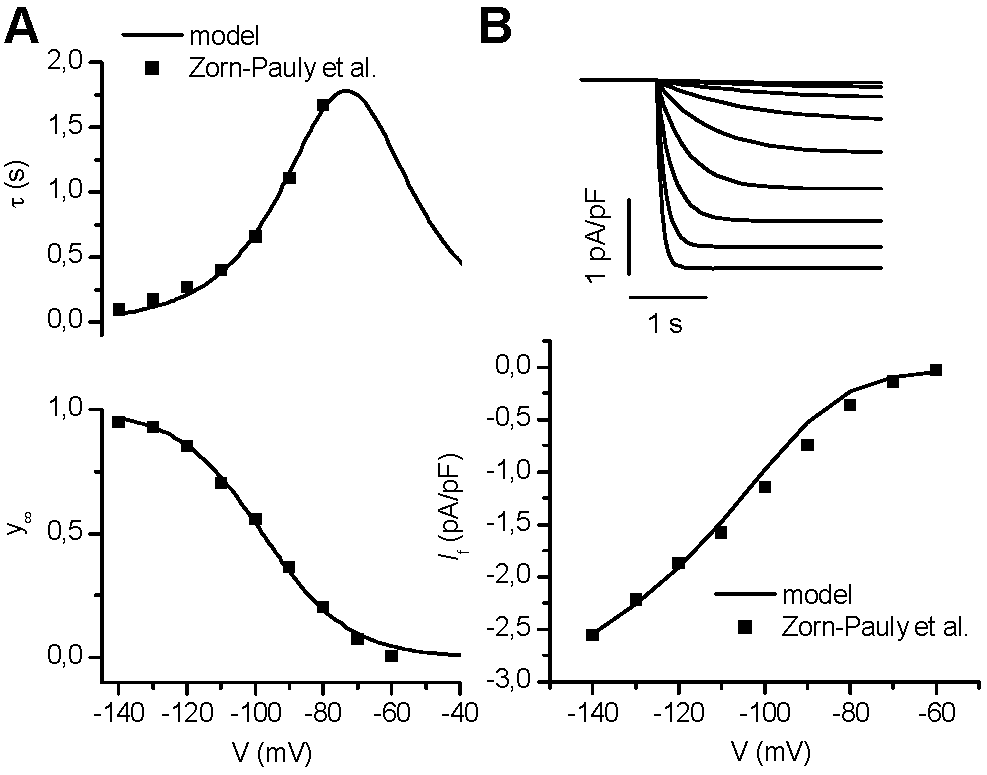

Supplement: Figure S2 — Steady-state activation and time-constant of If. Experimental conditions were replicated from [12]. The current traces of If were determined by application of hyperpolarizing voltage steps from −40 to −140 mV in 10 mV steps, the holding potential was −40 mV. The If model was fitted to the left atrium wall data from [12]. (0.07 MB TIF) [file pcbi.1001067.s002.tif]

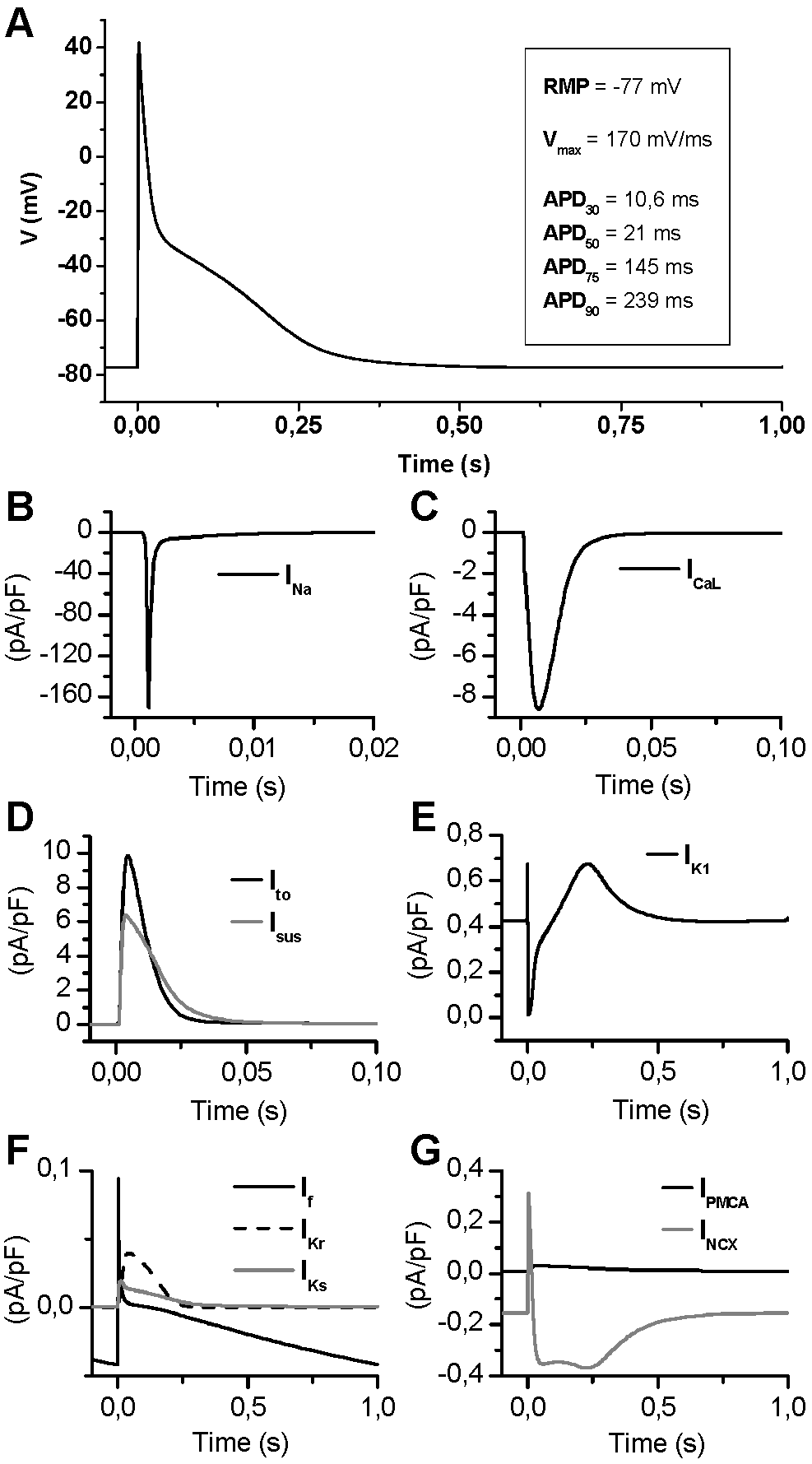

Supplement: Figure S3 — The principal outputs of the myocyte model at 1 Hz pacing. (A) The model reproduces an AP that is characteristic for human atrial myocytes: a large initial peak with a narrow early plateau is followed by a late, low amplitude, plateau phase; a so-called triangular shape. (B) and (C) The major depolarizing currents in the initial phase of the AP are the INa and ICaL. (D) The Ito (black line) and Isus (grey line) generate large repolarizing currents in the beginning of the AP. (E) The late repolarization is carried mostly by IK1 (F). As demonstrated by the time courses of If (black solid line), IKs (black dashed line), and IKr (grey line), they contribute very little to the AP, compared to Ito and Isus. (G) During the late phase of the AP, the INCX (grey line) generates a significant depolarizing current, while the amplitude of IPMCA (black line) is much smaller. (0.17 MB TIF) [file pcbi.1001067.s003.tif]
