# Supplementary material for: Impact of Sarcoplasmic Reticulum Calcium Release on Calcium Dynamics and Action Potential Morphology in Human Atrial Myocytes: A Computational Study
Source: PLoS Comput Biol. 2011 Jan 27;7(1):e1001067. doi: 10.1371/journal.pcbi.1001067 (PMC3029229; doi:10.1371/journal.pcbi.1001067)
Supplement: Table S1 — Modified parameter values of the previously published model components. (0.01 MB PDF) [file pcbi.1001067.s004.pdf]

Table S1. Modified parameter values of the previously published model components

Parameter	Definition	Criteria for modification [ref]	Value
P_{Na}	permeability of I_{Na}	Adjusting AP characteristics	0.00182 nL/s
g_{IK1}	conductance of I_{K1}	Adjusting end repolarization of AP and resting potential	3.825 nS
g_{ICab}	conductance of background calcium current	Adjusting diastolic $[Ca^{2+}]_i$	0.0952 nS
g_{INCX}	conductance of I_{NCX}	Adjusting calcium transient characteristics	0.008433945 pA/(mmol/L)^4
I_{CaPmax}	maximum pump rate of SL calcium pump	Adjusting diastolic $[Ca^{2+}]l_i$	2 pA
K_{mf}	SERCA half-maximal binding in cytosol	Adapted from [1]	0.00025 mM
K_{mr}	SERCA half-maximal binding in SR	Adapted from [1]	1.8 mM
k_4	pumping rate from SERCA to SR	Adapted from [2]	7.5 s^{-1}
c_{pumps}	concentration of SERCA	Based on [2] fitting of Ca^{2+} removal by SERCA	0.04 mM
k_{SRleak}	SR leak scaling parameter	Adjusting SR calcium content	0.006 s^{-1}

REFERENCES

1. Shannon TR, Ginsburg KS, Bers DM (2000) Reverse mode of the sarcoplasmic reticulum calcium pump and load-dependent cytosolic calcium decline in voltage-clamped cardiac ventricular myocytes. Biophysical Journal 78: 322-333.
2. Bers DM (2001) Excitation-contraction coupling and cardiac contractile force. Dordrecht ; Boston: Kluwer Academic Publishers. xxiv, 427 p. p.
