# Supplementary material for: Impact of Sarcoplasmic Reticulum Calcium Release on Calcium Dynamics and Action Potential Morphology in Human Atrial Myocytes: A Computational Study
Source: PLoS Comput Biol. 2011 Jan 27;7(1):e1001067. doi: 10.1371/journal.pcbi.1001067 (PMC3029229; doi:10.1371/journal.pcbi.1001067)
Supplement: Table S2 — Parameters of the novel RyR model. (0.01 MB PDF) [file pcbi.1001067.s005.pdf]

Table S2. Parameters of the novel RyR model

Parameter	Definition	Value
τ_{RyRact}	RyR activation time constant	0.01875 s (0.005 s in subspace)
$\tau_{RyRinact}$	RyR inactivation time constant	0.0875 s (0.015 s in subspace)
$\tau_{RyRadapt}$	RyR adaptation time constant	1 s
v	RyR maximum flux	1 s^{-1} (625 s^{-1} in subspace)
