# Supplementary material for: Impact of Sarcoplasmic Reticulum Calcium Release on Calcium Dynamics and Action Potential Morphology in Human Atrial Myocytes: A Computational Study
Source: PLoS Comput Biol. 2011 Jan 27;7(1):e1001067. doi: 10.1371/journal.pcbi.1001067 (PMC3029229; doi:10.1371/journal.pcbi.1001067)
Supplement: Table S3 — Comparison of AP characteristics of the developed model (vCa) and the two extended variants (vCaNass and vCaNassIk) to experiments. (0.01 MB PDF) [file pcbi.1001067.s006.pdf]

Table S3. Comparison of AP characteristics of the developed model (vCa) and the two extended variants (vCaNass and vCaNassIk) to experiments

Parameter	vCA	vCaNass	vCaNassIk	Experimental value [ref]
V_{diast} (mV)	-77.0	-76.0	-75.3	-79.9 ± 1.3 [1]; T = 36°C
				-75 ± 1 [2]; T = 35°C
				-68 ± 8 [3]; T = 37°C
V_{syst} (mV)	41.7	41.6	41.7	35 ± 2.8 [1]; T = 36°C
				53 ± 2 [4]; T = 37°C
V_{ampl} (mV)	118.8	117.7	117.0	98 ± 5 [5]; T = 36°C
				118.33 ± 3.00 [6]; T = 36°C
				130 ± 2 [4]; T = 37°C
dV/dt_{max} (mV/ms)	170	169	168	167 ± 26 [5]; T = 36°C
				231.9 ± 13.6 [6]; T = 36°C
				172 ± 60 [7]; T = 31°C
APD_{30} (ms)	10.6	10.8	10.8	13 ± 16 [7]; T = 31°C
				13.9 ± 3.7 [8]; T = 23°C; 0.5 Hz
APD_{50} (ms)	21.0	20.8	20.8	7.65 ± 1.12 [1]; T = 36°C
				62 ± 91 [7]; T = 31°C
				25 ± 3 [3]; T = 37°C
APD_{75} (ms)	145.4	148.4	136.0	141 ± 12 [9]; T = 37°C
APD_{90} (ms)	239.4	244.4	246.8	204 ± 11 [10]; T = 36°C
				237 ± 18 [9]; T = 37°C
				255 ± 45 [11]; T = 35°C

REFERENCES

1. Wang YG, Xu HY, Kumar R, Tipparaju SM, Wagner MB, et al. (2003) Differences in transient outward current properties between neonatal and adult human atrial myocytes. Journal of Molecular and Cellular Cardiology 35: 1083-1092.
2. Wettwer E, Hala O, Christ T, Heubach JF, Dobrev D, et al. (2004) Role of I-Kur in controlling action potential shape and contractility in the human atrium - Influence of chronic atrial fibrillation. Circulation 110: 2299-2306.
3. Dobrev D, Graf E, Wettwer E, Himmel HM, Hala O, et al. (2001) Molecular Basis of Downregulation of G-Protein-Coupled Inward Rectifying K^+ Current ($I_{K,ACh}$) in Chronic Human Atrial Fibrillation: Decrease in GIRK4 mRNA Correlates With

Reduced $I_{K,ACh}$ and Muscarinic Receptor-Mediated Shortening of Action Potentials. Circulation 104: 2551-2557.
4. Workman AJ, Kane KA, Russell JA, Norrie J, Rankin AC (2003) Chronic beta-adrenoceptor blockade and human atrial cell electrophysiology: evidence of pharmacological remodelling. Cardiovasc Res 58: 518-525.
5. Wang Z, Pelletier L, Talajic M, Nattel S (1990) Effects of flecainide and quinidine on human atrial action potentials. Role of rate-dependence and comparison with guinea pig, rabbit, and dog tissues. Circulation 82: 274-283.
6. Lagrutta A, Wang JX, Fermini B, Salata JJ (2006) Novel, potent inhibitors of human Kv1.5 K+ channels and ultrarapidly activating delayed rectifier potassium current. Journal of Pharmacology and Experimental Therapeutics 317: 1054-1063.
7. Dawodu AA, Monti F, Iwashiro K, Schiariti M, Chiavarelli R, et al. (1996) The shape of human atrial action potential accounts for different frequency-related changes in vitro. Int J Cardiol 54: 237-249.
8. Neef S, Dybkova N, Sossalla S, Ort KR, Fluschnik N, et al. CaMKII-Dependent Diastolic SR Ca^{2+} Leak and Elevated Diastolic Ca^{2+} Levels in Right Atrial Myocardium of Patients With Atrial Fibrillation. Circ Res 106: 1134-1144.
9. Pau D, Workman AJ, Kane KA, Rankin AC (2005) Electrophysiological effects of prucalopride, a novel enterokinetic agent, on isolated atrial myocytes from patients treated with beta-adrenoceptor antagonists. J Pharmacol Exp Ther 313: 146-153.
10. Dobrev D, Ravens U (2003) Remodeling of cardiomyocyte ion channels in human atrial fibrillation. Basic Res Cardiol 98: 137-148.
11. Bosch RF, Zeng XR, Grammer JB, Popovic K, Mewis C, et al. (1999) Ionic mechanisms of electrical remodeling in human atrial fibrillation. Cardiovasc Res 44: 121-131.
