# Supplementary material for: Impact of Sarcoplasmic Reticulum Calcium Release on Calcium Dynamics and Action Potential Morphology in Human Atrial Myocytes: A Computational Study
Source: PLoS Comput Biol. 2011 Jan 27;7(1):e1001067. doi: 10.1371/journal.pcbi.1001067 (PMC3029229; doi:10.1371/journal.pcbi.1001067)
Supplement: Table S4 — Initial values for the differential variables at 1 Hz pacing steady-state. (0.01 MB PDF) [file pcbi.1001067.s007.pdf]

Table S4. Initial values for the differential variables at 1 Hz pacing steady-state

Parameter	Definition	Value
V_m	membrane voltage (mV)	-77,1494
I_{Nam}	I_{Na} gating variables	0,00225
I_{Nah1}		0,92828
I_{Nah2}		0,92661
I_{Cald}	I_{CaL} gating variables	7,89E-06
I_{Calf1}		0,9991
I_{Calf2}		0,9991
I_{Calfca}		0,97263
I_{tr}	I_t gating variables	8,21E-04
I_{ts}		0,96044
I_{susr}	I_{sus} gating variables	1,11E-04
I_{suss}		0,99516
I_{Ksn}	I_{Ks} gating variable	0,00404
I_{Krpa}	I_{Kr} gating variable	3,16E-05
I_{fy}	I_f gating variable	0,06216
RyR_{oss}	values of RyR gating variables (open, closed, adaptation) in different compartments	5,14E-05
RyR_{css}		0,99994
RyR_{ass}		0,24361
RyR_{oc1}		1,01E-04
RyR_{cc1}		0,99932
RyR_{ac1}		0,19145
RyR_{oc2}		8,47E-05
RyR_{cc2}		0,99947
RyR_{ac2}		0,19984
RyR_{oc3}		6,47E-05
RyR_{cc3}		0,99955
RyR_{ac3}		0,21486
$[SERCACa^{2+}]_1$	concentrations of Ca^{2+} bound to SERCA in cytosolic	0,00458

$[SERCACa^{2+}]_2$	compartments (mM)	0,00446
$[SERCACa^{2+}]_3$		0,00428
$[SERCACa^{2+}]_{ss}$		0,00424
$[Na^+]_i$	cytosolic Na^+ concentration (mM)	9,43843
$[K^+]_i$	cytosolic K^+ concentration (mM)	134,366
$[Ca^{2+}]_{ss}$	Ca^{2+} concentrations in cytosolic compartments (mM)	1,68E-04
$[Ca^{2+}]_{c1}$		1,36E-04
$[Ca^{2+}]_{c2}$		1,40E-04
$[Ca^{2+}]_{c3}$		1,47E-04
$[Ca^{2+}]_{c4}$		1,61E-04
$[Ca^{2+}]_{SR1}$	Ca^{2+} concentrations in SR compartments (mM)	0,61372
$[Ca^{2+}]_{SR2}$		0,60256
$[Ca^{2+}]_{SR3}$		0,58564
$[Ca^{2+}]_{SR4}$		0,56883
