# Supplementary material for: Impact of Sarcoplasmic Reticulum Calcium Release on Calcium Dynamics and Action Potential Morphology in Human Atrial Myocytes: A Computational Study
Source: PLoS Comput Biol. 2011 Jan 27;7(1):e1001067. doi: 10.1371/journal.pcbi.1001067 (PMC3029229; doi:10.1371/journal.pcbi.1001067)
Supplement: Text S1 — Model implementation. (0.16 MB PDF) [file pcbi.1001067.s008.pdf]

Text S1: Model implementation and simulation protocols

Impact of sarcoplasmic reticulum calcium release on calcium dynamics and action potential morphology in human atrial myocytes: a computational study

Jussi T. Koivumäki, Topi Korhonen, Pasi Tavi

A.I. Virtanen Institute for Molecular Sciences
Department of Biotechnology and Molecular Medicine
University of Eastern Finland
Finland

Contents

Model equations	2
Ca^{2+} fluxes between SR and cytosol	2
Intracellular and SR Ca^{2+} dynamics	3
Sarcolemmal ion currents	6
Model variants	7
Simulation protocols	8
References	13

MODEL EQUATIONS

The presented mathematical model is a set of ordinary differential equations that were implemented to the Matlab™ (The MathWorks) environment of technical programming. Simulation results were obtained by numerically integrating the model equations with a stiff ordinary differential equation solver method (ode15s).

Except for the novel Ca^{+} dynamics as well as the new features and modifications described in the *Methods* section, the model equations are as in Nygren *et al.* [1] (see Tables 5-18 in the original ref.). Initial values for the differential variables are listed in Table S1.

Fluxes are in 1 nl volume. Note that cytosol compartments are 1, 2, 3, 4 and ss (subspace) and SR compartments are 1, 2, 3 and 4. The Ca^{2+} fluxes between SR and cytosol are as numbered in compartments 1-3, but Ca^{2+} flux from SR 4 goes to subspace (see Figure 1).

Ca^{2+} fluxes between SR and cytosol in compartments x = 1, 2, 3 and ss (1, 2, 3 and 4 for SR).

Serca

$$k_1 = 10^6 k_4 \tag{1}$$

$$k_2 = k_1 K_{mf}^2 \tag{2}$$

$$k_3 = \frac{k_4}{K_{mr}^2} \tag{3}$$

$$J_{SERCASRx} = \left\{ -k_3 [\mathrm{Ca}^{2+}]_{\mathrm{SRx}}^2 \left(c_{pumps} - [\mathrm{SERCACa}^{2+}]_x \right) + k_4 [\mathrm{SERCACa}^{2+}]_x \right\} V_x \tag{4}$$

$$J_{xSERCA} = \left\{ k_1 [\mathrm{Ca}^{2+}]_{\mathrm{ix}}^2 \left(c_{pumps} - [\mathrm{SERCACa}^{2+}]_x \right) - k_2 [\mathrm{SERCACa}^{2+}]_x \right\} V_x \tag{5}$$

$$\frac{d[SERCACa^{2+}]_x}{dt} = \frac{J_{xSERCA} - J_{SERCASRx}}{V_x} \tag{6}$$

RyR

$$RyR_{SRCax} = 1 - \frac{1}{1 + e^{\frac{[\mathrm{Ca}^{2+}]_{\mathrm{SR}x} - 0.3}{0.1}}} \tag{7}$$

$$RyR_{a\infty x} = 0.505 - \frac{0.427}{1 + e^{\frac{1000[\mathrm{Ca^{2+}}]_{\mathrm{i}x} - 0.29}{0.082}}} \tag{8}$$

$$RyR_{o\infty x} = 1 - \frac{1}{1 + e^{\frac{1000[\mathrm{Ca^{2+}}]_{\mathrm{i}x} - (RyR_{ax} + 0.22)}{0.03}}} \tag{9}$$

$$RyR_{c\infty x} = \frac{1}{1 + e^{\frac{1000[\mathrm{Ca^{2+}}]_{\mathrm{i}x} - (RyR_{ax} + 0.02)}{0.01}}} \tag{10}$$

$$J_{relx} = v_x RyR_{ox} RyR_{cx} RyR_{SRCax} \left([\mathrm{Ca^{2+}}]_{\mathrm{SR}x} - [\mathrm{Ca^{2+}}]_{\mathrm{i}x} \right) V_x \tag{11}$$

$$\frac{dRyR_{ax}}{dt} = \frac{RyR_{a\infty x} - RyR_{ax}}{\tau_{RyRadapt}} \tag{12}$$

$$\frac{dRyR_{ox}}{dt} = \frac{RyR_{o\infty x} - RyR_{ox}}{\tau_{RyRact}} \tag{13}$$

$$\frac{dRyR_{cx}}{dt} = \frac{RyR_{c\infty x} - RyR_{cx}}{\tau_{RyRinact}} \tag{14}$$

SR Ca^{2+} leak

$$J_{SRleakx} = k_{SRleak} \left([\mathrm{Ca^{2+}}]_{\mathrm{SR}x} - [\mathrm{Ca^{2+}}]_{\mathrm{i}x} \right) V_x \tag{15}$$

Intracellular and SR Ca^{2+} dynamics

Area accessible for diffusion between junctional and non-junctional cytosol

$$A_{j-nj} = 2\pi r_{junct} l_{cell} \times 0.5 \tag{16}$$

Distance between centers of junctional and $bulk_4$ compartment

$$x_{j-nj} = \frac{0.2}{2} + \frac{\Delta r}{2} \tag{17}$$

Ca^{2+} diffusion flux between junctional and non-junctional ($bulk_4$ compartment) cytosol

$$J_{j-nj} = D_{Ca} \frac{A_{j-nj}}{x_{j-nj}} \left([\mathrm{Ca^{2+}}]_{\mathrm{ss}} - [\mathrm{Ca^{2+}}]_{\mathrm{i4}} \right) \tag{18}$$

Intracellular Ca^{2+} fluxes

$$J_{Ca1} = -J_{1SERCA1} + J_{SRleak1} + J_{rel1} \tag{19}$$

$$J_{Ca2} = -J_{1SERCA2} + J_{SRleak2} + J_{rel2} \tag{20}$$

$$J_{Ca3} = -J_{1SERCA3} + J_{SRleak3} + J_{rel3} \tag{21}$$

$$J_{Ca4} = J_{j-nj} \tag{22}$$

$$J_{Cass} = -J_{j-nj} + J_{SRleakss} - J_{ssSERCA} + J_{relss} \tag{23}$$

SR Ca^{2+} fluxes

$$J_{SRCa1} = J_{SERCASR1} - J_{SRleak1} - J_{rel1} \tag{24}$$

$$J_{SRCa2} = J_{SERCASR2} - J_{SRleak2} - J_{rel2} \tag{25}$$

$$J_{SRCa3} = J_{SERCASR3} - J_{SRleak3} - J_{rel3} \tag{26}$$

$$J_{SRCa4} = J_{SERCASRss} - J_{SRleakss} - J_{relss} \tag{27}$$

Ca^{2+} buffers, x = 1,2,3 and 4

$$\beta_{ss} = \left[1 + \frac{[\mathrm{SLlow}]K_{dSLlow}}{\left([\mathrm{Ca}^{2+}]_{ss} + K_{dSLlow}\right)^2} + \frac{[\mathrm{SLhigh}]K_{dSLhigh}}{\left([\mathrm{Ca}^{2+}]_{ss} + K_{dSLhigh}\right)^2} + \frac{[\mathrm{BCa}]K_{dBCa}}{\left([\mathrm{Ca}^{2+}]_{ss} + K_{dBCa}\right)^2}\right]^{-1} \tag{28}$$

$$\beta_{ix} = \left[1 + \frac{[\mathrm{BCa}]K_{dBCa}}{\left([\mathrm{Ca}^{2+}]_{\mathrm{ix}} + K_{dBCa}\right)^2}\right]^{-1} \tag{29}$$

$$\gamma_{ix} = \frac{[\mathrm{BCa}]K_{dBCa}}{[\mathrm{Ca}^{2+}]_{\mathrm{ix}} + K_{dBCa}} \tag{30}$$

$$\beta_{SRx} = \left[1 + \frac{[\mathrm{CSQN}]K_{dCSQN}}{\left([\mathrm{Ca}^{2+}]_{\mathrm{SRx}} + K_{dCSQN}\right)^2}\right]^{-1} \tag{31}$$

Intracellular Ca^{2+} concentration

$$\frac{d[\mathrm{Ca}^{2+}]_{ss}}{dt} = \beta_{ss}\left(\frac{J_{Cass}}{V_{ss}} + \frac{-I_{CaL} - I_{Cab} - I_{CaP} + 2I_{NaCa}}{2V_{ss}F}\right) \tag{32}$$

For the simulation of Ca^{2+} in bulk cytosol the general equation

$$\frac{\partial[\mathrm{Ca}^{2+}]_\mathrm{i}}{\partial t} = \beta_i(D_{Ca} + \gamma_i D_{CaBm})\nabla^2[\mathrm{Ca}^{2+}]_\mathrm{i} - \frac{2\beta_i\gamma_i D_{CaBm}}{K_{dBCa} + [\mathrm{Ca}^{2+}]_\mathrm{i}}\nabla[\mathrm{Ca}^{2+}]_\mathrm{i}\cdot\nabla[\mathrm{Ca}^{2+}]_\mathrm{i} + J_{Ca}\beta_i \tag{33}$$

is approximated with radial symmetry to following ODEs:

$$\boldsymbol{j} = [1\ 2\ 3\ 4] \tag{34}$$

for x = 1

$$\frac{d[\mathrm{Ca}^{2+}]_\mathrm{ix}}{dt} = \beta_{ix}(D_{Ca} + \gamma_{ix}D_{CaBm})\left(\frac{[\mathrm{Ca}^{2+}]_\mathrm{ix+1} - [\mathrm{Ca}^{2+}]_\mathrm{ix}}{\Delta r^2} + \frac{[\mathrm{Ca}^{2+}]_\mathrm{ix+1} - [\mathrm{Ca}^{2+}]_\mathrm{ix}}{2j_x\Delta r^2}\right) - \frac{2\beta_{ix}\gamma_{ix}D_{CaBm}}{K_{dBCa} + [\mathrm{Ca}^{2+}]_\mathrm{ix}} \times \left(\frac{[\mathrm{Ca}^{2+}]_\mathrm{ix+1} - [\mathrm{Ca}^{2+}]_\mathrm{ix}}{2\Delta r}\right)^2 + \frac{J_{Cax}}{V_x}\beta_{ix} \tag{35}$$

for x = 2, 3

$$\frac{d[\mathrm{Ca}^{2+}]_\mathrm{ix}}{dt} = \beta_{ix}(D_{Ca} + \gamma_{ix}D_{CaBm})\left(\frac{[\mathrm{Ca}^{2+}]_\mathrm{ix+1} - 2[\mathrm{Ca}^{2+}]_\mathrm{ix} + [\mathrm{Ca}^{2+}]_\mathrm{ix-1}}{\Delta r^2} + \frac{[\mathrm{Ca}^{2+}]_\mathrm{ix+1} - [\mathrm{Ca}^{2+}]_\mathrm{ix-1}}{2j_x\Delta r^2}\right) - \frac{2\beta_{ix}\gamma_{ix}D_{CaBm}}{K_{dBCa} + [\mathrm{Ca}^{2+}]_\mathrm{ix}} \times \left(\frac{[\mathrm{Ca}^{2+}]_{ix+1} - [\mathrm{Ca}^{2+}]_{ix-1}}{2\Delta r}\right)^2 + \frac{J_{Cax}}{V_x}\beta_{ix} \tag{36}$$

for x = 4

$$\frac{d[\mathrm{Ca}^{2+}]_\mathrm{ix}}{dt} = \beta_{ix}(D_{Ca} + \gamma_{ix}D_{CaBm})\left(\frac{-[\mathrm{Ca}^{2+}]_{\mathrm{i}x} + [\mathrm{Ca}^{2+}]_{\mathrm{i}x-1}}{\Delta r^2} + \frac{[\mathrm{Ca}^{2+}]_{\mathrm{i}x} - [\mathrm{Ca}^{2+}]_{\mathrm{i}x-1}}{2j_x\Delta r^2}\right) - \frac{2\beta_{ix}\gamma_{ix}D_{CaBm}}{K_{dBCa} + [\mathrm{Ca}^{2+}]_\mathrm{ix}} \times \left(\frac{[\mathrm{Ca}^{2+}]_{\mathrm{i}x} - [\mathrm{Ca}^{2+}]_{ix-1}}{2\Delta r}\right)^2 + \frac{J_{Cax}}{V_x}\beta_{ix} \tag{37}$$

Similarly, for the simulation of Ca^{2+} in SR the general equation

$$\frac{\partial[\mathrm{Ca}^{2+}]_\mathrm{SR}}{\partial t} = \beta_{SR}D_{CaSR}\nabla^2[\mathrm{Ca}^{2+}]_\mathrm{SR} + J_{SRCa}\beta_{SR} \tag{38}$$

is approximated with radial symmetry to following ODEs:

for x = 1

$$\frac{d[\mathrm{Ca}^{2+}]_{\mathrm{SRx}}}{dt} = \beta_{SRx} D_{CaSR} \left(\frac{[\mathrm{Ca}^{2+}]_{\mathrm{SR}x+1} - [\mathrm{Ca}^{2+}]_{\mathrm{SR}x}}{\Delta r^2} + \frac{[\mathrm{Ca}^{2+}]_{\mathrm{SR}x+1} - [\mathrm{Ca}^{2+}]_{\mathrm{SR}x}}{2 j_x \Delta r^2} \right) + \frac{J_{SRCax}}{V_{SRx}} \beta_{SRx} \tag{39}$$

for x = 2, 3

$$\frac{d[\mathrm{Ca}^{2+}]_{\mathrm{SRx}}}{dt} = \beta_{SRx} D_{CaSR} \left(\frac{[\mathrm{Ca}^{2+}]_{\mathrm{SR}x+1} - 2[\mathrm{Ca}^{2+}]_{\mathrm{SR}x} + [\mathrm{Ca}^{2+}]_{\mathrm{SR}x-1}}{\Delta r^2} + \frac{[\mathrm{Ca}^{2+}]_{\mathrm{SR}x+1} - [\mathrm{Ca}^{2+}]_{\mathrm{SR}x-1}}{2 j_x \Delta r^2} \right) + \frac{J_{SRCax}}{V_{SRx}} \beta_{SRx} \tag{40}$$

for x = 4

$$\frac{d[\mathrm{Ca}^{2+}]_{\mathrm{SRx}}}{dt} = \beta_{SRx} D_{CaSR} \left(\frac{-[\mathrm{Ca}^{2+}]_{\mathrm{SR}x} + [\mathrm{Ca}^{2+}]_{\mathrm{SR}x-1}}{\Delta r^2} + \frac{[\mathrm{Ca}^{2+}]_{\mathrm{SR}x} - [\mathrm{Ca}^{2+}]_{\mathrm{SR}x-1}}{2 j_x \Delta r^2} \right) + \frac{J_{SRCax}}{V_{SRx}} \beta_{SRx} \tag{41}$$

Accesible volumes of cytosol and SR compartments

$$V_x = \pi l_{cell} [(j_x \Delta r)^2 - (j_{x-1} \Delta r)^2] \times 10^{-6} \times 0.5 \tag{42}$$

$$V_{SRx} = 0.0225 V_x \tag{43}$$

Sarcolemmal ion currents

The SL ion currents were mostly formulated as in the previously published model of human atrial AP [1]. All the major modifications and novel features of the model are described below. Minor adjustments of parameter values are listed in Table S1. I_{to}, I_{sus}, I_{Ks}, I_{Kr}, I_{Nab} and I_{NaK} were implemented as in [1].

Ca^{2+} current

The I_{CaL} was formulated as

$$I_{\mathrm{CaL}} = g_{\mathrm{CaL}} d f_{ca} f_1 f_2 (V - E_{Ca,app}) \tag{44}$$

The time-constant for gate f_1 was fitted to the same data as previously [1,2]. However, as shown in Figure S1A, it was adjusted to have larger values in the membrane voltage range

of -40 and -10 mV; an approach that has been used in other models as well [3]. This modification of the I_{CaL} formulation was necessary to reduce the overly large tendency of the model to produce early after-depolarizations due to re-activation of I_{CaL}. The steady-state curve for the Ca^{2+}-dependent inactivation gate was formulated as

$$f_{ca,\infty} = 1 - \frac{1}{1 + \left(\frac{0.001}{[\mathrm{Ca}^{2+}]_{\mathrm{i,ss}}}\right)^2} \tag{45}$$

The time-constant for $f_{ca,\infty}$ was adopted from [4]; see Figure S1B, upper panel. As shown in the lower panel of Figure S1B, this formulation reproduces qualitatively the result that blocking intracellular Ca^{2+} release decreases the rate of L-type calcium channel inactivation significantly in human atrial myocytes [5]. The channel conductance, g_{CaL}, was adjusted to 25.3125 nS to reproduce a -12 pA/pF peak current [6,7,8] (Figure S1C) and proper Ca^{2+} intrusion via L-type Ca^{2+} channels [5]. See Results for further details.

Hyperpolarization-activated inward K^+ current

The hyperpolarization-activated inward current (I_f) was not included in the SL currents of the original Nygren model. Recent experiments indicate that it could have a significant role in atrial myocytes [9,10,11,12], so it was added according to a previously published formulation [12]. The curves for steady-state activation and time-constant were fitted to experimental data [12], as shown in Figure S2. The current equations were implemented according to the formulation of Zorn-Pauly et al. [12] (see Chapter 2.3 for equations in the original ref).

MODEL VARIANTS

To compare the role of SR Ca^{2+} dynamics in the modulation of action potential duration (APD) to other recently suggested mechanisms, we implemented two additional variants of the myocyte model that included:

(1) a subsarcolemmal Na^+ compartment and

(2) an updated scheme of K^+ currents (I_{to} and I_{sus}).

The description of the subsarcolemmal ‘fuzzy’ space for Na^+ corresponds to the recent *in vitro* findings from ventricular myocytes [13,14,15,16]. The Na^+ diffusion coefficient and buffering capacity were set according to the estimates of Despa et al. [15] and Grandi et al. [17], respectively. The size of the subsarcolemmal ‘fuzzy’ space is the same as the corresponding Ca^{2+} compartment. The area accessible for diffusion between junctional and cytosolic compartments, the distance between centers of junctional and cytosolic

compartment, and the Na^+ diffusion flux between the junctional and cytosolic compartment are calculated as for Ca^{2+} in equations (16–18).

The K^+ ion currents (I_{to} and I_{sus}) were updated according to a recently published human atrial model of Maleckar et al. [18]. The implemented is based on the description in the appendix of the original publication.

In both model variants, #1 and #2, the maximum conductance of inwardly rectifying K^+ current (g_{K1}) was decreased by 4% and 10%, respectively, to adjust APD_{90} at 1Hz pacing to a comparable level.

Simulation protocols

As suggested originally by Hund et al. [19], we implemented a conservative stimulation scheme, in which the stimulus current is carried by K^+ ions. In pacing experiments, the normal stimulus was a rectangular current pulse that was repeated according to the pacing frequency.

In quiescent experiments, one stimulus pulse was applied at the beginning of the simulation and the myocyte was then left undisturbed for a time period that was required for the model to reach a steady-state.

REFERENCES

1. Nygren A, Fiset C, Firek L, Clark JW, Lindblad DS, et al. (1998) Mathematical model of an adult human atrial cell - The role of K^+ currents in repolarization. Circ Res 82: 63-81.
2. Li GR, Nattel S (1997) Properties of human atrial I_{Ca} at physiological temperatures and relevance to action potential. Am J Physiol Heart Circ Physiol 41: H227-H235.
3. ten Tusscher KHWJ, Panfilov AV (2006) Alternans and spiral breakup in a human ventricular tissue model. American Journal of Physiology-Heart and Circulatory Physiology 291: H1088-H1100.
4. ten Tusscher KHWJ, Noble D, Noble PJ, Panfilov AV (2004) A model for human ventricular tissue. American Journal of Physiology-Heart and Circulatory Physiology 286: H1573-H1589.
5. Hatem SN, Benardeau A, RuckerMartin C, Marty I, deChamisso P, et al. (1997) Different compartments of sarcoplasmic reticulum participate in the excitation-contraction coupling process in human atrial myocytes. Circulation Research 80: 345-353.
6. Nygren A, Fiset C, Firek L, Clark JW, Lindblad DS, et al. (1998) Mathematical model of an adult human atrial cell - The role of K+ currents in repolarization. Circulation Research 82: 63-81.
7. Li GR, Nattel S (1997) Properties of human atrial I-Ca at physiological temperatures and relevance to action potential. American Journal of Physiology-Heart and Circulatory Physiology 41: H227-H235.
8. Ouadid H, Albat B, Nargeot J (1995) Calcium Currents in Diseased Human Cardiac-Cells. Journal of Cardiovascular Pharmacology 25: 282-291.
9. Hoppe UC, Beuckelmann DJ (1998) Characterization of the hyperpolarization-activated inward current in isolated human atrial myocytes. Cardiovasc Res 38: 788-801.
10. Porciatti F, Pelzmann B, Cerbai E, Schaffer P, Pino R, et al. (1997) The pacemaker current I_f in single human atrial myocytes and the effect of b-adrenoceptor and A_1-adenosine receptor stimulation. Br J Pharmacol 122: 963-969.
11. El Chemaly A, Magaud C, Patri S, Jayle C, Guinamard R, et al. (2007) The Heart Rate-Lowering Agent Ivabradine Inhibits the Pacemaker Current I_f in Human Atrial Myocytes. J Cardiovasc Electrophysiol 18: 1190-1196.
12. Zorn-Pauly K, Schaffer P, Pelzmann B, Lang P, Machler H, et al. (2004) I_f in left human atrium: a potential contributor to atrial ectopy. Cardiovasc Res 64: 250-259.
13. Wendt-Gallitelli MF, Voigt T, Isenberg G (1993) Microheterogeneity of Subsarcolemmal Sodium Gradients. Electron Probe Microanalysis in Guinea-Pig Ventricular Myocytes. J Physiol 472: 33-44.
14. Fujioka Y, Matsuoka S, Ban T, Noma A (1998) Interaction of the Na^+-K^+ pump and Na^+-Ca^{2+} exchange via $[Na^+]_i$ in a restricted space of guinea-pig ventricular cells. J Physiol 509: 457-470.
15. Despa S, Bers DM (2003) Na/K pump current and $[Na]_i$ in rabbit ventricular myocytes: Local $[Na]_i$ depletion and Na buffering. Biophys J 84: 4157-4166.
16. Verdonck F, Mubagwa K, Sipido KR (2004) $[Na^+]$ in the subsarcolemmal 'fuzzy' space and modulation of $[Ca^{2+}]_i$ and contraction in cardiac myocytes. Cell Calcium 35: 603-612.
17. Grandi E, Pasqualini FS, Puglisi JL, Bers DM (2009) A Novel Computational Model of the Human Ventricular Action Potential and Ca transient. Biophys J 96: 664a-665a.
18. Maleckar MM, Greenstein JL, Giles WR, Trayanova NA (2009) K^+ current changes account for the rate dependence of the action potential in the human atrial myocyte. Am J Physiol Heart Circ Physiol 297: H1398-1410.

19. Hund TJ, Kucera JP, Otani NF, Rudy Y (2001) Ionic charge conservation and long-term steady state in the Luo-Rudy dynamic cell model. Biophys J 81: 3324-3331.
